# Nitrogen Fixation and Microbial Communities Associated with Decomposing Seagrass Leaves in Temperate Coastal Waters

**DOI:** 10.1007/s00248-024-02424-w

**Published:** 2024-08-14

**Authors:** Vasiliki Papazachariou, Victor Fernández-Juárez, Laura Wegener Parfrey, Lasse Riemann

**Affiliations:** 1 Marine Biological Section, Department of Biology, University of Copenhagen, Helsingør, Denmark; 2 Center for Volatile Interactions, Department of Biology, University of Copenhagen, Copenhagen, Denmark; 3 Biodiversity Research Centre, Department of Botany, and Department of Zoology, University of British Columbia, Vancouver, Canada

**Keywords:** Nitrogen fixation, Diazotrophs, Decomposition, *Zostera marina*, *nifH*, 16S rRNA

## Abstract

Seagrass meadows play pivotal roles in coastal biochemical cycles, with nitrogen fixation being a well-established process associated with living seagrass. Here, we tested the hypothesis that nitrogen fixation is also associated with seagrass debris in Danish coastal waters. We conducted a 52-day in situ experiment to investigate nitrogen fixation (proxied by acetylene reduction) and dynamics of the microbial community (16S rRNA gene amplicon sequencing) and the nitrogen fixing community (*nifH* DNA/RNA amplicon sequencing) associated with decomposing *Zostera marina* leaves. The leaves harboured distinct microbial communities, including distinct nitrogen fixers, relative to the surrounding seawater and sediment throughout the experiment. Nitrogen fixation rates were measurable on most days, but highest on days 3 (dark, 334.8 nmol N g^−1^ dw h^−1^) and 15 (light, 194.6 nmol N g^−1^ dw h^−1^). Nitrogen fixation rates were not correlated with the concentration of inorganic nutrients in the surrounding seawater or with carbon:nitrogen ratios in the leaves. The composition of nitrogen fixers shifted from cyanobacterial *Sphaerospermopsis* to heterotrophic genera like *Desulfopila* over the decomposition period. On the days with highest fixation, *nifH* RNA gene transcripts were mainly accounted for by cyanobacteria, in particular by *Sphaerospermopsis* and an unknown taxon (order Nostocales), alongside Proteobacteria. Our study shows that seagrass debris in temperate coastal waters harbours substantial nitrogen fixation carried out by cyanobacteria and heterotrophic bacteria that are distinct relative to the surrounding seawater and sediments. This suggests that seagrass debris constitutes a selective environment where degradation is affected by the import of nitrogen via nitrogen fixation.

**Supplementary Information:**

The online version contains supplementary material available at 10.1007/s00248-024-02424-w.

## Introduction

Seagrass meadows are among the most productive marine ecosystems [1, 2]. Seagrasses are angiosperms thriving underwater, contributing to primary production through their photosynthesis while offering important ecosystem services such as shore protection, sediment stabilization and biodiversity enhancement [3, 4]. Seagrass production is exported to the surroundings as particulate and dissolved organic matter affecting carbon cycling on local and global scales [5, 6].

The regulation of nutrient cycling and retention within seagrass meadows occurs through both direct processes involving uptake and assimilation in leaves, roots and rhizomes, as well as indirect mechanisms such as the trapping of organic matter present in suspended particles [7–10]. Nitrogen may limit seagrass productivity, especially in oligotrophic environments [11], and can be supplied through nitrogen fixation associated with aboveground parts such as seagrass leaves, or belowground like roots and rhizosphere. For instance, nitrogen fixation in the rhizosphere can meet nearly all of the plant’s nitrogen requirements, and this assimilated nitrogen can subsequently be transported to the aboveground tissues [10, 12]. Studies on nitrogen fixation associated with leaves, especially in temperate waters, are few [13–16], but report significant and variable rates associated with epiphytes on *Zostera marina* leaves [17].

Seagrass debris represents a substantial biomass in some coastal waters and its degradation by microbes affects local carbon (C), nitrogen (N), phosphorus (P), sulphur and iron cycling [18–20]. Seagrass debris is characterized by rather high C:N:P ratios and leaves have a higher C:N ratio than rhizomes due to a high cellulose content [18], making decomposition slow [21]—slower than other marine litter, such as macroalgal detritus [22]. During macroalgal decomposition, labile nitrogen is preferentially utilized by microbes compared to carbon, likely leading to N limitation, which in turn might be alleviated by diazotrophic activity [23, 24]. Faster utilization of detrital carbon accelerates the macroalgal degradation, while N limitation may hinder microbial processes and slow macroalgal decomposition rates [24]. Indeed, in detrital macroalgal systems, nitrogen enrichment was connected to microbial proliferation [25] and nitrogen fixation appeared stimulated by declining C:N ratios during the decomposition process [26]. Moreover, nitrogen fixation rates were much higher than observed in association with living macroalgae [26]. Extensive microbial colonization and decomposition of seagrass debris is well-known [18, 27, 28]; however, it is not known whether and to what extent diazotrophs are involved. This is important because nitrogen fixation could influence debris decomposition [29] and the associated elemental cycling and represent a hitherto overlooked N input to coastal systems [17].

In this study, we examined nitrogen fixation and the diazotroph community associated with debris of the eelgrass *Z. marina*. This species thrives in Danish temperate waters [30], although it has declined during the past century due to eutrophication [31], and plays a fundamental role in coastal ecosystems throughout the northern hemisphere [32, 33]. To our knowledge, it is not known whether nitrogen fixation is associated with debris of *Z. marina* leaves, but in Danish waters rates of nitrogen fixation were about three times higher in vegetated sediments in comparison with non-vegetated ones [34]. We, therefore, hypothesized that decomposing eelgrass leaves are foci for nitrogen fixation. Such N import could affect the degradation of eelgrass as well as the cycling of nutrients and carbon in this coastal environment. We expected that the environmental changes occurring during the course of leaf degradation, including C, N and P contents, and nutrient availability in the surrounding seawater, would affect nitrogen fixation and be mirrored in successional changes in the associated communities of heterotrophic and phototrophic bacteria. We specifically addressed the early phases of seagrass decomposition, including the anticipated passive leaching and microbial colonization phases. To address this and determine the relative importance of phototrophic and heterotrophic diazotrophs, we measured nitrogen fixation associated with decomposing seagrass leaves under light and dark conditions over 52 days. In parallel, we explored the associated microbial community composition and dynamics with a specific focus on diazotroph communities.

## Materials and Methods

### Experimental Design and Sampling

Seagrass shoots were collected in July 2022 from a *Z. marina* meadow in a semi-enclosed bay by Helsingør, Denmark, via freediving (depth < 2 m, 56°2′9.82″N, 12°36′48.96″E). The site was chosen because it harbours a healthy meadow typical for these coastal waters. Ten fresh shoots were added to each of 11 polyester 1.0-mm mesh bags (Hydro-Bios Apparatebau GmbH) (30 × 30 cm) that were then randomly attached to a 2 × 2 m metallic grid at ~ 2-m depth, on bare sediment, roughly 30 m from the meadow (Fig. S1). All bags were placed ca. 5 cm above the sediment. Temperature and light were continuously monitored by loggers attached to the grid (HOBO® MX2202 Data Logger, Onset Computer Corp., Bourne, MA, USA). Between 9 and 10 am, on days 0, 1, 3, 4, 7, 10, 15, 22, 36, 43 and 52, one bag was removed and transported to the laboratory in a bucket with ambient seawater. Within 15 min of sampling, leaves were fixed for subsequent RNA extraction or transferred to serum vials for ARA measurements (see below).

At each time point, biomass for microbial community composition analysis was sampled from surrounding seawater and seagrass leaves (~ 8–10 cm sections) in triplicates. Seagrass samples were immediately preserved in 1 ml RNAlater (Thermo Fisher Scientific, MA, USA) in 1.5 ml Eppendorf tubes, and stored at − 20 °C. Surrounding seawater was sampled from within the metallic grid using sterile 5-l plastic bags. Seawater (500 ml) was filtered in triplicates onto Durapore® filters (0.22 µm, 25 mm diameter, Sigma-Aldrich, MA, USA), which were stored at − 20 °C. The filtrate was stored in − 20 °C in duplicates in 15 ml Falcon tubes and further analysed for ammonium (NH_4_^+^), nitrate (NO_3_^2−^) and phosphate (PO_4_^3−^). NH_4_^+^ was quantified fluorometrically [35]. NO_3_^2−^ and PO_4_^3−^ were quantified using standard colorimetric methods [36, 37]. On days 10, 15, 22, 36, 43 and 52, top sediment, to 5-cm depth, was sampled using a 15-ml Falcon tube from within the grid area. On days 0, 15 and 43, seawater was sampled from within the mesh bags using a 500 ml syringe and then filtered and stored for subsequent community analysis (see below).

### Measurements of Nitrogen Fixation

Nitrogen fixation was estimated using acetylene reduction assay (ARA) as described earlier [16, 37]. Despite the identified pitfalls of ARA as a method, it is the most commonly used method used in studies estimating nitrogen fixation associated with seagrass [15, 17, 39]. Seagrass leaves were randomly collected from the bag at each time point, cut into 10–12-cm-long pieces, and placed in 20 ml serum vials containing 1 ml of 0.2 µm filtered seawater from the sampling site. The vials were then sealed with crimped septa and 2 ml of acetylene (ALPHAGAZ ™ ACETYLEN ≥ 99.6%) was added to the gas phase using a gastight Hamilton syringe to obtain a 10% vol./vol. concentration. For each timepoint, measurements were performed for six seagrass replicates and two controls—light and dark. Due to seagrass fragmentation, only a dark incubation was carried out on day 52. Dark incubations were wrapped in aluminium foil. For the controls, a vial with 1 ml of 0.2 µm filtered seawater and no seagrass leaf controlled for abiotic ethylene production [40] whereas a vial with leaf but without acetylene added controlled for ethylene produced by the leaf. Any detection of ethylene production measured in the seawater controls (abiotic) was subtracted from the respective set of samples. In the seagrass set of controls (biotic), no ethylene production was measurable. The vials were incubated in situ at 1 m depth between 11 am and 5 pm. Light and temperature were recorded with a Pendant MX Temperature/Light Data Logger. Incubations were terminated when 10 ml of gas was transferred from the incubated vial to a 20 ml vacuumed and crimped serum vial with a gastight Hamilton syringe. Samples were kept at room temperature and measured the next day using a Shimadzu GC-2010 gas chromatograph with a flame ionization detector and a SS Porapak T column (2 mm) with a mesh range 80/100. To extrapolate ARA data (acetylene reduced to ethylene) to fixed N, a conversion factor of 3.9 was used as has previously been used for decomposing litter [41]. After incubation, the seagrass leaves were dried at 60 °C for 48 h and ground to powder. Each sample was weighed and packed in aluminium pockets for C and N analyses on a EuroVevtor (EuroEA_Elemental analyzer) and P on a FiaStar (FIAstar_5000 Analyzer). C:N ratio was calculated in mole C/g leaf and mole N/g leaf, respectively. The complete dataset consists of %C and %N.

### DNA and RNA Extractions

All DNA extractions from seagrass (51 samples), seawater (54 samples) and sediment (18 samples) and RNA extractions from seagrass (6 samples) were carried out in a laboratory where no *nifH* gene amplification work had been performed. DNA from seagrass (8–10 cm leaf sections) and sediment (1 g per sample) was extracted using the DNeasy PowerSoil Pro Kit (Qiagen, Hilden, Germany) following the manufacturer’s instructions. All samples were eluted in 10 mM Tris buffer (pH 8) and concentrations quantified using the Qubit™ 1 × dsDNA High Sensitivity (ThermoFisher, MA, USA). DNA from seawater was extracted from Durapore® membrane filters using the DNeasy Power Soil Pro Kit, after grinding the filters into powder using a sterilized metallic grinder. Seagrass leaf RNA was extracted using the AllPrep DNA/RNAMini Kit (Qiagen Sciences, MD, USA) according to the manufacturer’s instructions with the modification that the cell lysis step with β-mercaptoethanol, RLT Plus, included zirconium beads from the DNeasy PowerSoil Pro Kit and bead beading (benchtop vortex with bead tube adapter, max speed, 10 min, room temperature). Extracted RNA was quantified (Qubit™ RNA High Sensitivity, ThermoFisher, MA, USA) and stored at − 70 °C.

### cDNA Synthesis and Amplicon Generation and Sequencing

cDNA was synthesized using Invitrogen™ SuperScript™ IV Reverse Transcriptase (Thermo Fischers Scientific, Invitrogen™, MA, USA), the nifH3 reverse primer [42] and 5 μL of RNA extract. Reverse transcriptase-free control reactions were included for all samples to verify complete DNA removal during RNA extraction. Amplicons of *nifH* were generated in triplicates in a nested PCR [43]. These primers capture a broad range of both cyanobacterial and non-cyanobacterial diazotrophs [44]. The PCR reactions (25 μl) contained 12.5 μL MyTaq™ HS Mix 2 × DNA Polymerase (Bioline Reagents) and 0.4 μM forward and reverse primers. Initial denaturation was at 94 °C for 120 s, followed by 30 cycles of 60 s at 94 °C, 60 s at 54 °C, 60 s at 72 °C and a final 420 s at 72 °C. PCR triplicates were pooled, size confirmed by agarose gel electrophoresis and purified (MP Biomedicals™ Geneclean™ Turbo Kit). Amplicon libraries were Illumina indexed (National Genomics Infrastructure, Uppsala, Sweden), purified (Beckman Coulter™ Agencourt AMPure XP, ThermoFisher, MA, USA), quantified (Qubit™ 1X dsDNA High Sensitivity), pooled in equimolar ratios and sequenced using an Illumina MiSeq platform (2 × 300 bp pair-end reads, GeoGenetics Sequencing Core, University of Copenhagen, Denmark). Negative controls of PCR UV-irradiated water were included for each PCR reaction and DNA extraction round; negative controls were checked by gel electrophoresis and never generated visible amplification. These control samples for PCR (a total of eight) and DNA extractions (a total of six) were pooled in two respective pools and sequenced to account for potential background contamination. The read numbers from controls never exceeded 1% of the average read number obtained from samples (54,168 reads per sample) and were judged not to influence the data.

### 16S rRNA gene amplification

The V3-V4 hypervariable region of the 16S rRNA gene was PCR amplified using the primers 341F/805R (341F, 5′-CCTACGGGNGGCWGCAG-3′; 805R, 5′-GACTACHVGGGTATCTAATCC-3′) [45]. Initial denaturation was at 94 °C for 180 s, followed by 30 cycles of 45 s at 94 °C, 60 s at 50 °C, 90 s at 72 °C and a final 90 s at 72 °C. All samples were performed in triplicates. The size of the amplicons was confirmed, and they were further purified using MP Biomedicals™ Geneclean™ Turbo Kit (ThermoFisher, MA, USA). An amplicon library was constructed with specific barcodes assigned to each sample and then barcoded amplicons were cleaned (Beckman Coulter™ Agencourt AMPure XP). Finally, amplicons were quantified (Qubit™ 1 × dsDNA High Sensitivity; ThermoFisher, MA, USA), pooled in equimolar ratios and sequenced using an Illumina MiSeq platform with 2 × 300 bp pair-end reads (GeoGenetics Sequencing Core, Copenhagen, Denmark).

### Data Analysis and Statistics

All analyses were performed in RStudio (version 4.2.2). Visualization of data was done with ggplot2 [46] and the Brewer colour palettes. Demultiplexing and trimming of indexes and adaptors were performed by the sequencing facility. Amplicon sequence variants (ASVs) were generated using the DADA2 pipeline [47] yielding lengths of 325–328 bp (*nifH* gene) and 360–368 bp (16S RNA gene). A total of 10,535 *nifH* ASVs and 4531 16S rRNA ASVs were obtained from the respective 93 and 30 samples. An average number of 500 reads was observed in the controls and no ASV was assigned to any of the controls after trimming and denoising of the sequences. Sequences were processed with the phyloseq package [48] and after evaluating the rarefaction curves samples with < 1000 reads were removed (Fig. S2). For the *nifH* analysis: one replicate from seagrass days 0 and 4, respectively. For the 16S rRNA gene analysis: one replicate from seagrass day 3. Based on the rarefaction curves for the 16S rRNA gene and *nifH* analysis, alpha and beta diversity measures data were rarefied to depths of 10,000 and 20,000 reads, respectively. For *nifH*, six samples were removed since they contained fewer reads than the threshold (day 0 seagrass replicate 1, day 4 seagrass replicates 1 and 2, day 24 seagrass replicate 1 and day 36 seawater replicate 1) and for 16S rRNA genes one sample day 15 seagrass replicate 1. *NifH* taxonomy was assigned with the ‘assignTaxonomy’ from the DADA2 pipeline, using a *nifH* reference database (v. June 2017, Zehr lab). 16S rRNA gene ASV taxonomy was assigned according to the SILVA database (silva_nr_v132_train_set). The Shannon diversity index [49] was calculated based on normalized data (rarefied to even depth) and compared across decomposition days with Wilcoxon Rank sum test followed by Bonferroni correction for multiple comparisons. For non-metric multidimensional scaling (NMDS), Bray–Curtis distance was chosen as the dissimilarity measure (*nifH* and 16S rRNA genes). Permutational multivariate analysis of variance (two-factor PERMANOVA) was conducted to assess statistical differences in community composition using the ‘adonis2’ function applying 999 permutations in ‘vegan’ package (v.2.6–4 [50]). Data on nitrogen fixation were tested for normal distribution between different groups using Levene’s test (*P* < 0.0001) and statistical significance was tested using non-parametric Kruskal–Wallis followed by Dunn’s post hoc test for pairwise comparisons. Statistical significance between days was tested with a non-parametric Kruskal–Wallis (*χ*^2^ = 56.56, *P*_adj_ < 0.001) followed by a post hoc Dunn test using Bonferroni correction. Differences on C:N ratios between different days were tested with analysis of variance (ANOVA) followed by a Tukey’s honest significant difference (HSD) to identify significant pairwise comparisons.

Phylogenetic analysis was used to determine the phylogenetic affiliations of the nine ASVs from the *nifH* DNA and RNA datasets assigned as UCYN-A (*Candidatus* Atelocyanobacterium thalassa)*.* Nucleotide sequences of these ASVs and 14 reference sequences were aligned using MUSCLE 5 [51] in Geneious Prime (v.2024.0.2). UCYN-A nucleotide reference sequences were from [52–54], and the remaining reference sequences were acquired from NCBI [55]. Then RaxML 8.2.11 was used to construct a maximum-likelihood tree with 1000 bootstraps under the GTR GAMMA model [56].

Sequences have been deposited in the NCBI GenBank database with accession numbers SAMN41318939-SAMN41319031 classified under the Bio Project number PRJNA1110172.

## Results

### Dynamics in Nitrogen Fixation and C, N and P Contents During the Decomposition of Seagrass Leaves

During the decomposition period, seawater temperature varied from 18 °C to 21.9 °C (Fig. S3). Concentrations of nitrate (NO_3_^2−^) and phosphate (PO_4_^3−^) in the surrounding seawater varied from 0.35 to 5.88 μM and from 0.16 to 0.45 μM, respectively, and showed peak concentrations on days 7 and 22 (Fig. S4). Similarly, ammonia (NH_4_^+^) was highest on day 7 (0.12 μM; Fig. S4).

**Fig. 1:**
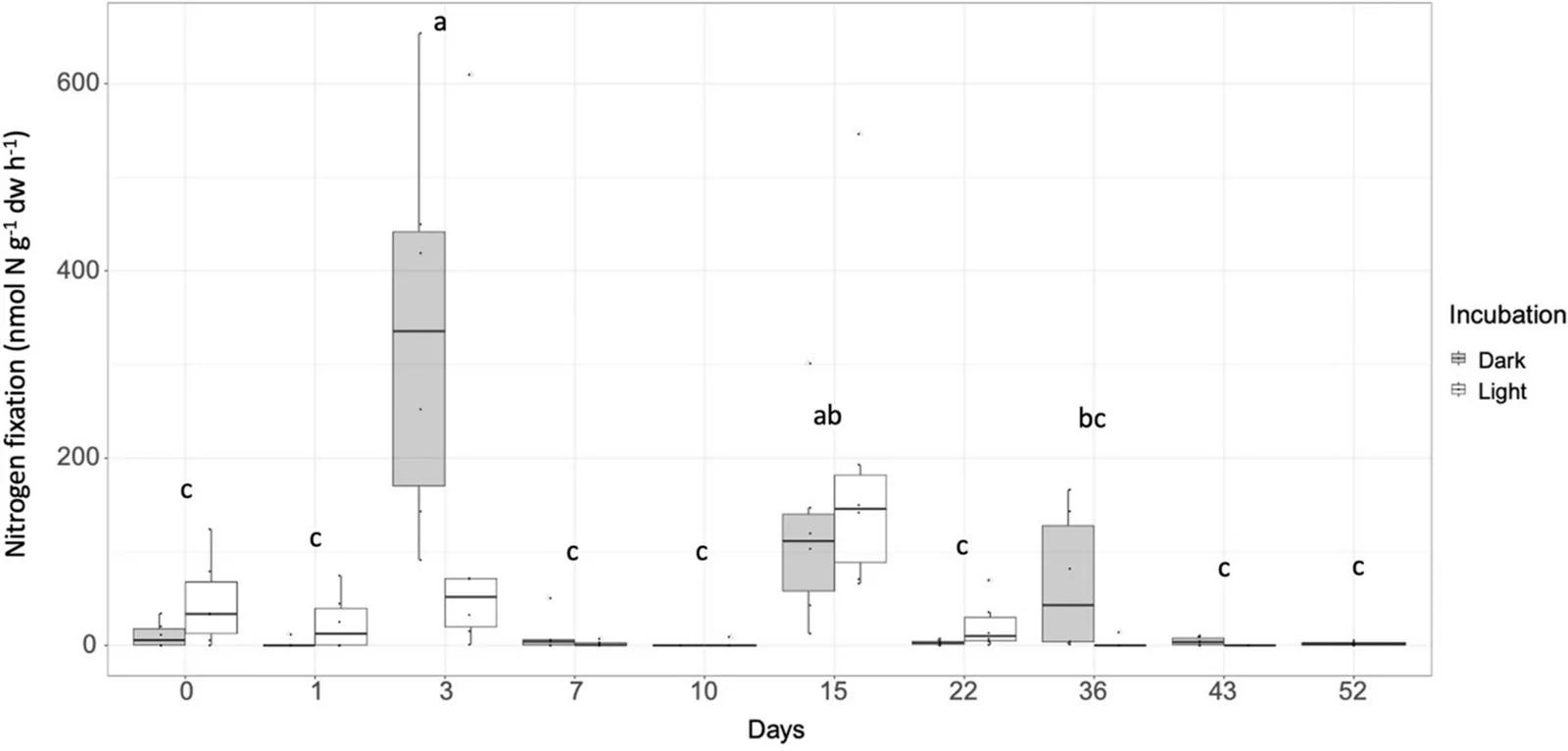
Nitrogen fixation rates associated with decomposing *Z. marina* leaves over time. Leaves (*n* = 6) were incubated under dark (dark grey) and light (white) conditions. The lines in the boxes represent the median. Letters above each bar represent statistical difference (*P*_adj_ < 0.05) between means of the days (light and dark incubations/day) tested with Kruskal–Wallis followed by a posthoc Dunn’s using Bonferroni correction

**Fig. 2:**
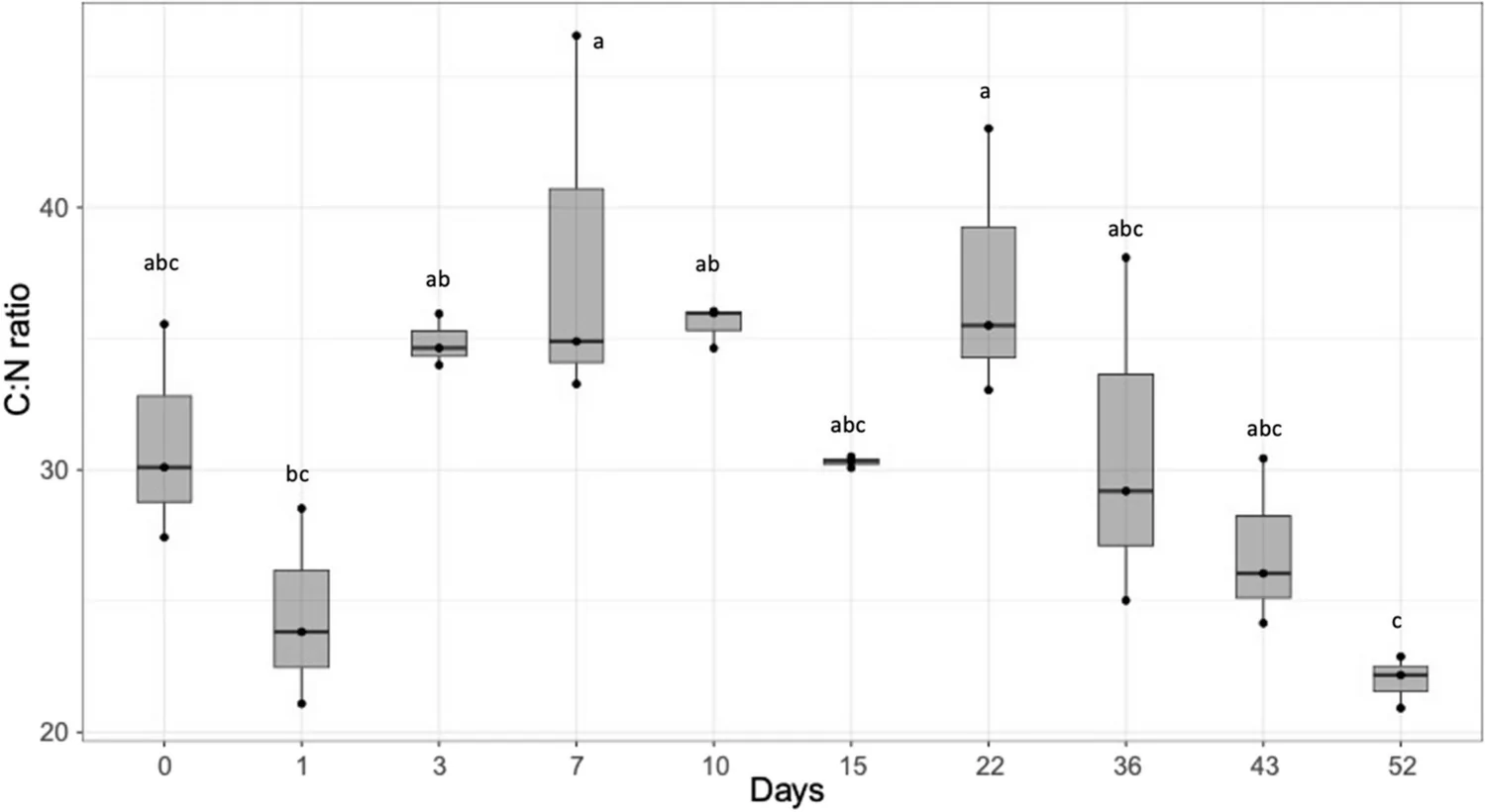
Carbon (C) to Nitrogen (N) ratios (C:N) of decomposing *Z. marina* leaves. Letters above each bar represent statistical difference (*P* < 0.05) between means tested with analysis of variance (ANOVA) followed by a post hoc Tukey test (‘TukeyHSD’)

Nitrogen fixation rates were measurable on most days. The highest rates were observed on days 3 (dark, 334.8 nmol N g^−1^ dw h^−1^) and 15 (light, 194.6 nmol N g^−1^ dw h^−1^; Fig. 1) (Kruskal–Wallis, df = 18, *P*_adj_ < 0.001). On days 3 and 36, rates under dark conditions were significantly higher than in the light (*P*_adj_ < 0.05) and on day 0 rates were higher in the light (*P*_adj_ = 0.04) (Fig. S5). The C:N ratio fluctuated between 25 and 35 and declined to a low of 21.9 on day 52 (C:N ratio on day 52 was significantly lower than on days 3, 7, 10 and 22; ANOVA, *P* < 0.001; Fig. 2). The P elemental leaf content did not change over the decomposition period (ANOVA, *P* < 0.05; Fig. S6). Nitrogen fixation under dark and light conditions was not correlated with any of the measured environmental parameters, including nutrient levels in the seawater or seagrass leaves (Fig. S7).

### Microbial Community Composition on Decomposing Seagrass Leaves

**Fig. 3:**
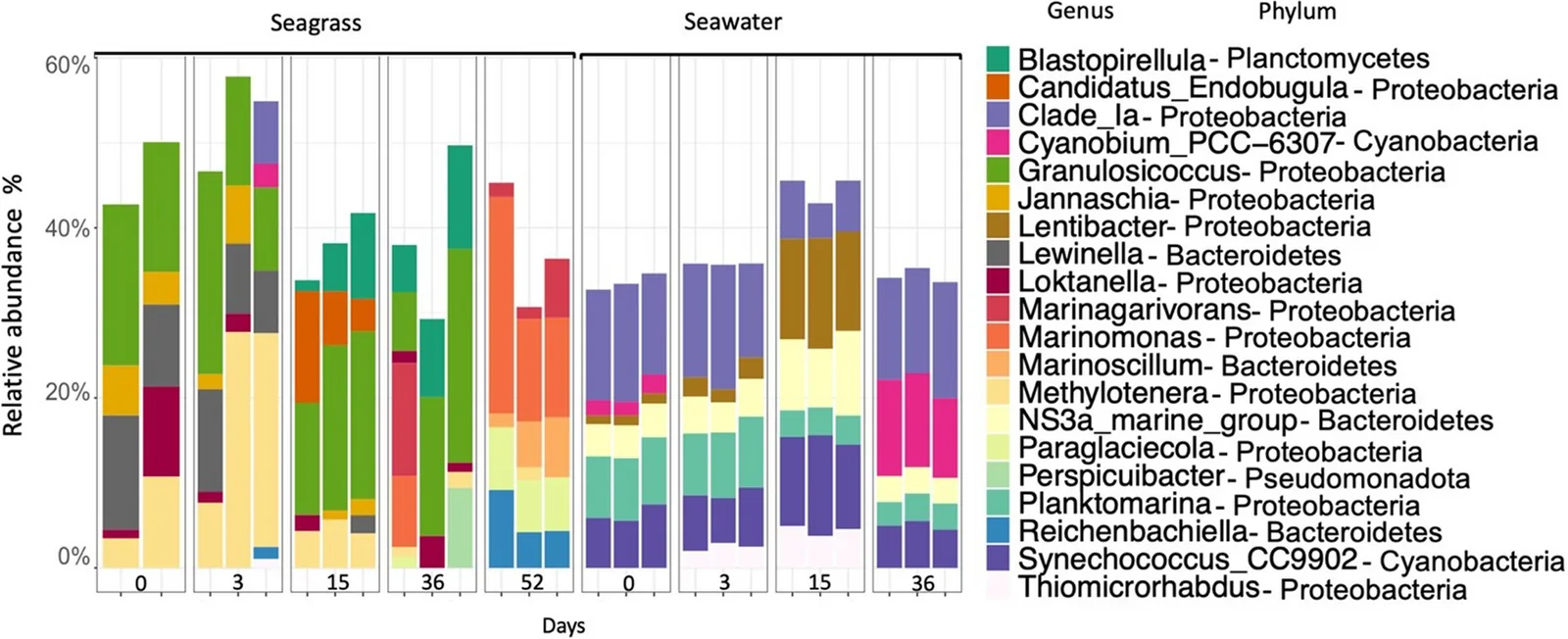
Microbial community composition on seagrass leaves and in the surrounding seawater based on 16S rRNA gene amplicon sequencing. The 20 most dominant Phyla and genera are shown

The microbial community composition on decomposing seagrass leaves, as analysed by 16S rRNA gene amplicon sequencing, was distinct from the surrounding seawater and showed a pronounced temporal succession (Fig. 3, Fig. S8). Sample type (seagrass versus seawater) explained 45% of the variation in microbial communities (PERMANOVA, *R*^2^ = 0.45, *P* = 0.001) while time explained 31% of the variation (PERMANOVA, *R*^2^ = 0.31, *P* = 0.01). Turnover in bacterial community composition on seagrass was evident in the most abundant genera (Fig. 3) and families over time (Fig. S9). The genera *Lewinella* (family Saprospiraceae) and *Granulosicoccus* (family Thiohalorhabdaceae) were initially abundant (accounting for 10 to 20% of relative abundance) and declined as decomposition progressed (Fig. 3). The genus *Blastopirellula* (family Pirellulaceae) peaked on days 15–36, accounting for up to 10% relative abundance (Fig. 3). On day 52, these taxa were replaced by *Marinomonas* (family Marinomonadaceae) and *Reichenbachiella* (family Cyclobacteriaceae).

**Fig. 4:**
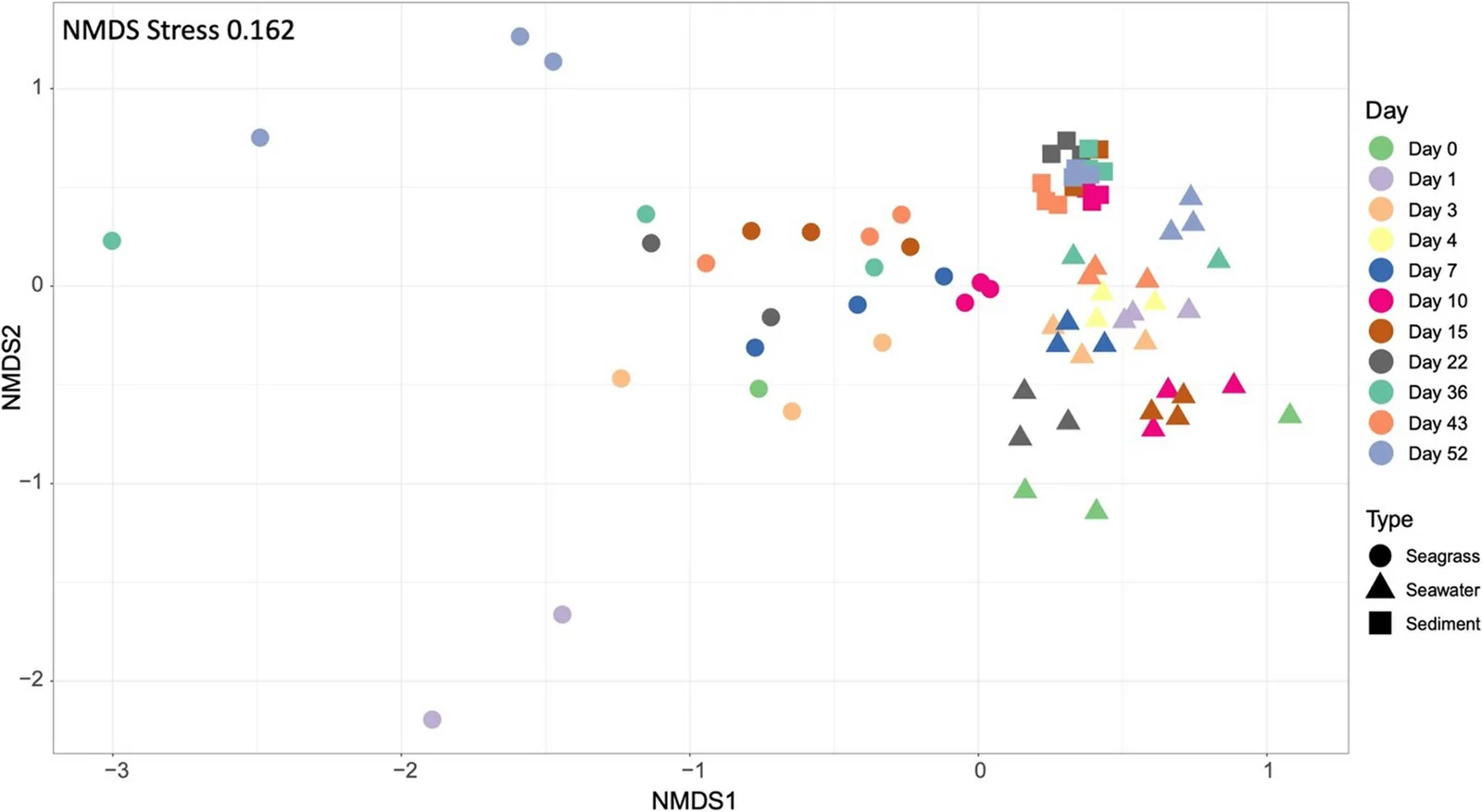
Non-metric multidimensional scaling (NMDS) of Bray–Curtis dissimilarity of *nifH* gene amplified from DNA samples from seagrass, seawater and sediment over the course of the decomposition experiment. Days are indicated by colour and substrate by shape

**Fig. 5:**
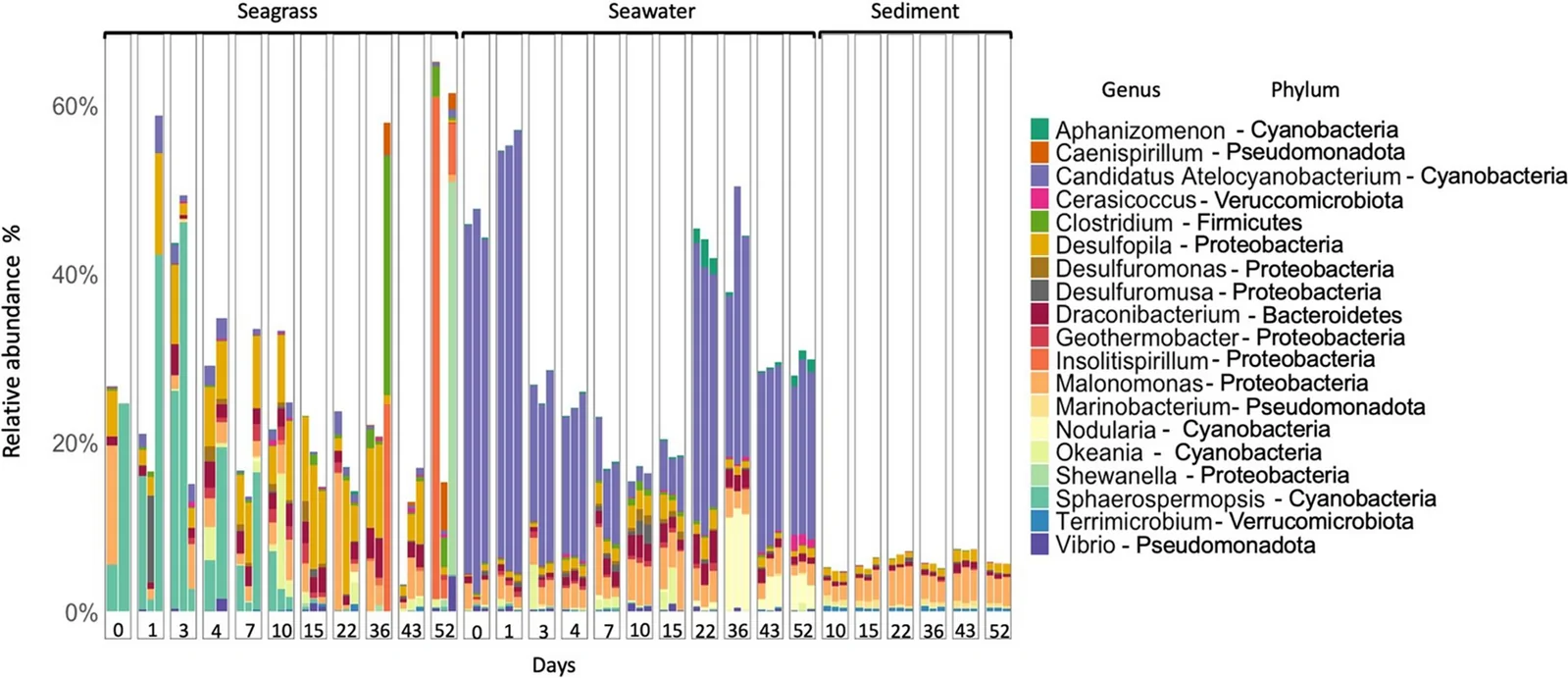
Composition of diazotrophs on seagrass, in surrounding seawater and in sediments over time in relative abundance. Based on *nifH* amplicon sequencing of DNA. Each bar represents one sample replicate. Only the 20 most dominant genera are shown. Sediment sampling started on day 10. One seagrass replicate from each of days 0 and 4 were excluded from the analysis due to low read numbers

The composition and succession of diazotrophs was analysed by *nifH* gene amplicon sequencing. The diazotrophs on seagrass showed a distinct composition relative to diazotrophs in the surrounding seawater and in sediment, with sample type explaining 40% of the variation (PERMANOVA, *R*^2^ = 0.40, *P* = 0.001; Fig. 4). Communities also changed over time, with decomposition day explaining 21% of the variation (PERMANOVA, *R*^2^ = 0.21, *P* < 0.001). Occasional water sampling with a syringe from within the incubation bags, and subsequent *nifH* gene sequencing, showed that the composition of free-living diazotrophs did not differ from diazotrophs in surrounding seawater (Fig. S10); i.e. the incubation in a bag did not select for a distinct community. The α-diversity of diazotrophs increased over the first 10 days and then leveled off (Fig. S11). The diazotrophs on seagrass belonged mainly to the classes Gammaproteobacteria, Cyanophyceae, Verrucomicrobiae, Bacteroidia and Deltaproteobacteria (Fig. S12). Cyanobacteria were consistently present throughout the incubation but declined gradually from 25% on day 1 to 5% on day 36 (mean of replicates). There was a notable increase on day 43, with cyanobacteria accounting for 39% of relative abundance, before declining to 5% on day 52 (data not shown). A deeper analysis on the genus level showed an extensive succession on seagrass with a change from a prevalence of the filamentous heterocyst forming cyanobacterium *Sphaerospermopsis* (family Aphanizomenonaceae) during the first 10 days to the emergence of mainly heterotrophic genera, like *Desulfopila* (days 7–43), and *Insolitispirillum* (Fig. 5). On the first 3–4 days, *Sphaerospermopsis* accounted for 12–25% (mean of replicates) of the relative abundance, but then gradually disappeared as the community became dominated by heterotrophic taxa. *Desulfopila* was relatively constant over time accounting for 3–10% (mean of replicates) of the total seagrass community with a peak on day 15. *Insolitispirillum* was only present from day 10 (0.1%) with an increase on day 36 (8.5%) and on day 52 where it accounted for 22.5% (mean of replicates) of the diazotrophs. Similarly, *Shewanella* was present on days 15, 36 and 52 only, and accounted for 16% (mean of replicates) on day 52. Diazotrophs in the surrounding seawater were dominated by different symbiotic unicellular cyanobacteria (UCYN-A; Candidatus Atelocyanobacterium Thalassa; class Cyanophyceae) (20–50% relative abundance) and in sediments by *Malonomonas* (2–4% relative abundance)*.* Six ASVs from seawater were affiliated with the UCYN-A2 and one with the UCYN-A4 sublineages, respectively (Fig. S13). Malonomonas is an anaerobic, microaerotolerant sediment bacterium [57]. Only one UCYN-A ASV was found in DNA from seagrass samples, and it showed > 95% nucleotide similarity to the six seawater UCYN-A ASVs affiliated with the UCYN-A2 sublineage.

**Fig. 6:**
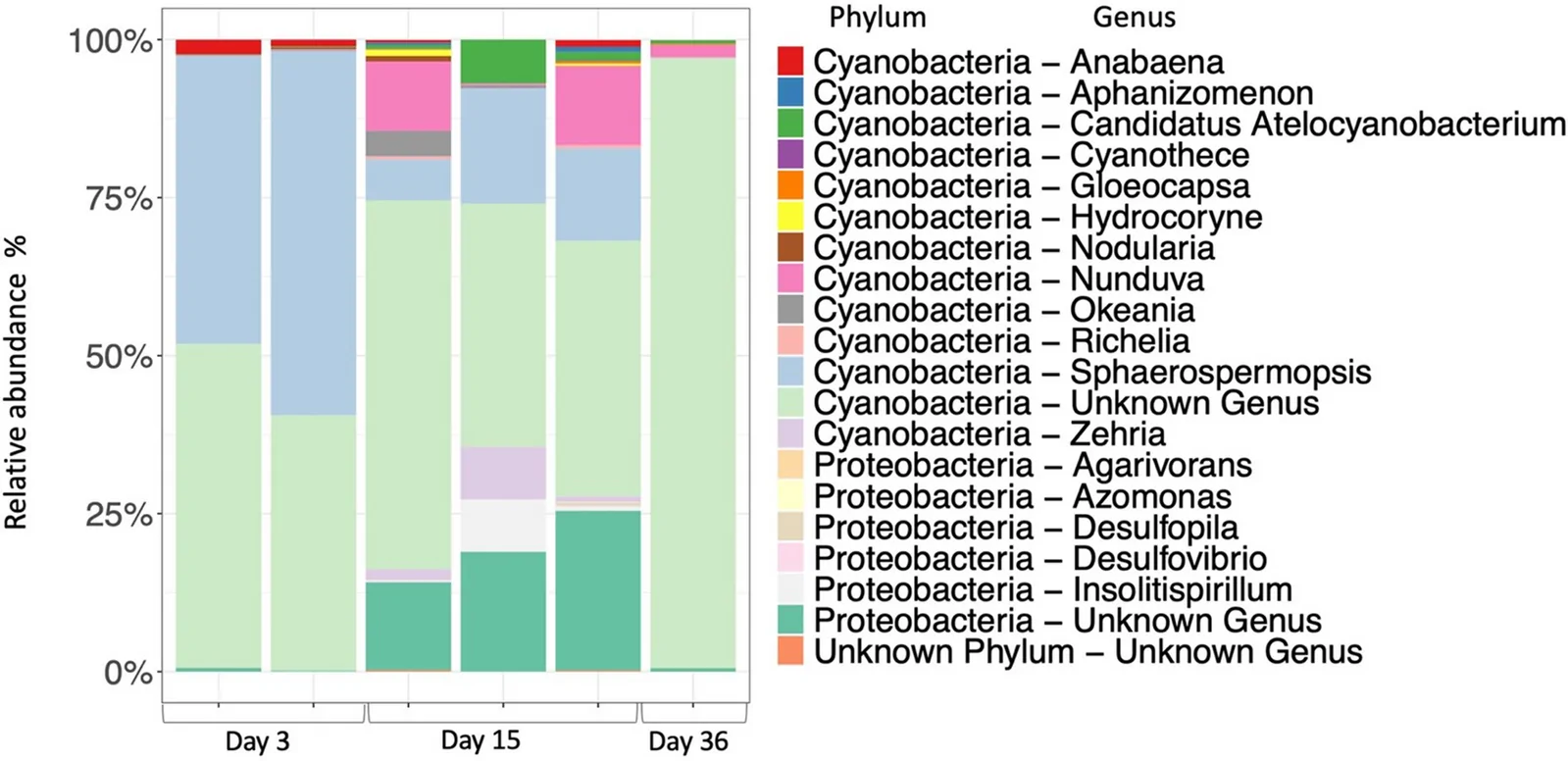
Relative abundance of the top 20 diazotrophs expressing nitrogenase (*nifH* RNA gene expression) on the 3 days with highest nitrogen fixation. Each bar represents one replicate

The organisms responsible for nitrogen fixation on the seagrass leaves were identified by sequencing *nifH* RNA on the days of peak nitrogen fixation (3, 15 and 36). The *nifH* RNA gene transcripts were dominated by Cyanobacteria throughout and the taxa with the highest transcript abundance varied between days and across replicates (Fig. 6). The cyanobacterial transcripts were partly accounted for by two ASVs of an unknown genus. They belonged to the order Nostocales and showed 91–92% nucleotide similarity with the Richelia cyanobiont of the diatom Rhizosolenia sp. (HQ586597). On day 3, the *nifH* gene transcripts were dominated by the cyanobacteria *Sphaerospermopsis* and *Anabaena*. On day 3, *Sphaerospermopsis* was responsible for 52% of the *nifH* gene transcripts (mean of amplifiable replicates) decreasing to 13% on day 15 and being almost absent on the last day (0.03%). On day 15, more diverse diazotrophs expressed *nifH*, including cyanobacteria such as *Sphaerospermopsis* and *Candidatus* Atelocyanobacterium thalassa (UCYN-A2) representing 13% and 3% of the transcripts and the heterocystous *Nunduva* (8%), but also with a presence of *Insolitispirillum* (3%), a purple nonsulphur bacterium from the family Rhodospirillaceae. The non-cyanobacterial *nifH* transcripts were also partially accounted for by one proteobacterial ASV of unknown genus (17%—mean of replicates) and showed 84.4% nucleotide similarity with *Azotobacter salinestris* strain CP045302. On day 36, *nifH* gene transcripts were identifiable from *Nunduva* (2%), *Candidatus* Atelocyanobacterium (UCYN-A2) (0.6%), *Gloeocapsa* (0.05%) and the heterotrophic bacterium *Agarivorans* (0.1%).

## Discussion

We found that nitrogen fixation is associated with decomposition of the seagrass *Z. marina* in Danish coastal waters and that the microbes responsible are distinct in composition relative to adjacent water and sediment environments. Our study indicates that seagrass leaves represent a selective environment inhabited by specialized microbes carrying out local nitrogen fixation.

### Nitrogen Fixation Associated with Decomposing Seagrass Leaves Is an Overlooked Nitrogen Input to Danish Coastal Waters

We measured nitrogen fixation rates up to 335 nmol N g^−1^ dw h^−1^ on decaying seagrass leaves. To our knowledge, these are the first rates reported for *Z. marina* leaf litter from temperate waters. They are comparable to rates measured on mangrove and macroalgal detritus: up to 380 nmol N g^−1^ dw h^−1^ [59] and 693 nmol N g^−1^ dw h^−1^ [26], respectively, and exceed rates measured on living seagrass leaves [16, 17, 60]. Higher levels of nitrogen fixation on debris relative to live plants seem to be a consistent observation, as it has also been observed for macroalgae [26] and mangrove leaf litter [59], possibly due to labile carbon availability during the degradation process [6, 61]. *Z. marina* is widespread in Denmark [30] and in the northern hemisphere [31, 32]. The high rates of nitrogen fixation associated with decaying seagrass leaves reported here suggest that they represent a nitrogen source to Danish coastal environments, which is hitherto unaccounted for.

To gain insight into the metabolism of active diazotrophs during the seagrass decomposition, nitrogen fixation was measured under dark and light conditions. Surprisingly, the dynamics appeared rather inconsistent with no significant overall difference for light and dark incubations, except for days 3 and 36, where rates were highest in the dark and day 0 where rates were highest in the light (Fig. 1). While the data from specific days are hard to explain, we suggest that the pattern of mainly fixation in the light in the first part of the degradation and the predominance of dark fixation after day 22 reflects the compositional succession from prevalence of phototrophic cyanobacteria (nitrogen fixation in the light) to heterotrophic bacteria (nitrogen fixation in the dark) when oxygen conditions are conceivably low on the degrading leaves [62]. The distinction between light and dark fixation is conceivably not clear-cut. Although many diazotrophic cyanobacteria fix nitrogen in the light [63, 64], others do also in the dark [72, 74] and heterotrophic diazotrophs may also do fixation in the light, and even exploit light [66]. Still, we note that an initial cyanobacterial fixation in light, supported by cyanobacterial *nifH* gene expression (Fig. 6), is consistent with the idea that this is partially driven by a pre-existing epiphytic community on fresh leaves, as proposed earlier [17, 26, 67, 68], and partially by acquired diazotrophs due to the decomposition process. Similarly, in early stages of macroalgal decomposition, nitrogen fixation activity under light conditions was associated with cyanobacterial epiphytes [26]. In general, nitrogen fixation associated with living seagrass leaves in temperate regions vary considerably, ranging from significantly higher rates under light conditions contributing nearly 95% of the total daily rate [17], to no discernible differences between light and dark nitrogen fixation [68], and to 99% of the total fixation occurring under dark conditions [67]. Hence, cyanobacteria likely dominate fixation on seagrass leaves during early degradation, whereas heterotrophic fixation becomes more important in the latter part of the degradation process. The persistent nitrogen fixation throughout the decomposing period under dark conditions, particularly after 22 days when rates were only measurable in the dark, suggests a contribution by heterotrophic diazotrophs. We speculate that this is linked to the establishment of an epiphytic biofilm on seagrass leaves, indicated by a decreased C:N ratio, leading to reduced oxygen availability [69–71], which could favour nitrogenase activity by heterotrophic bacteria. Still, it is noteworthy that the *nifH* gene expression is dominated by cyanobacteria, even at day 36 (see below and Fig. 6) suggesting they cyanobacteria continue to be active members of the epiphytic biofilm community.

### Factors Regulating Nitrogen Fixation During Seagrass Decomposition Are Unclear

We predicted that nitrogen fixation rates on seagrass detritus would be correlated with environmental nutrient concentrations and/or the C:N:P nutrient content of seagrass leaves. These predictions were informed by nutrient dynamics in macroalgal and macrophyte detrital systems [26, 59, 72, 73], the distinct phases of seagrass decomposition and the associated microbial activity [61], and the factors known to regulate nitrogen fixation [13, 74, 75]. However, no correlations between nitrogen fixation rates and the measured environmental parameters were found. We expected that the high C:N ratios and high availability of labile C, characteristic for fresh seagrass leaves, would stimulate nitrogen fixation early in decomposition, as observed for some macroalgal detrital systems [26]. We also predicted that C:N ratios would decrease later in decomposition due to the development of a microbial biofilm and an increased microbial biomass [26, 29, 76]. Our fresh seagrass leaves had a mean C:N ratio of 30 and the C:N ratios trended lower on day 1 (mean 23) and then increased to roughly 35 for days 3–22 before declining to a mean of 22 on day 52. However, there was no correlation between C:N ratios or P content of leaves and nitrogen fixation rates (Fig. S7), which were highest on days 3 and 15 (Fig. 1). We speculate that the observed nutrient dynamics result from a complex interplay between decomposition of the seagrass leaves and the formation of a microbial biofilm on the decomposing leaves—including both autotrophs and heterotrophs, utilizing nutrients in the seagrass while also fixing C and N [26, 29, 61].

### Composition and Temporal Succession of the Microbes Associated with Decomposing Seagrass Leaves

The microbial community associated with decomposing seagrass leaves was distinct from the microbial community found in nearby seawater and sediment. This is consistent with earlier findings on both live seagrass and during decomposition [20, 29, 61, 77–79] but in contrast with other studies that report overlap in microbial community composition between seagrass and surrounding seawater [80–82]. Here, we show that the composition of the diazotroph community was also distinct from seawater and sediment throughout decomposition. The microbial community associated with seagrass leaf decomposition consisted mainly of δ-proteobacteria, Cyanophyceae, γ-proteobacteria, α-proteobacteria, Planctomycetes and Bacteroidia. γ-proteobacteria and α-proteobacteria showed a notable increase in both datasets (*nifH* and 16S rRNA) over the course of decomposition. Several clades of γ-proteobacteria and α-proteobacteria, recognized as marine copiotrophs [83], are proposed as indicators of active leaf decomposition [20]. Moreover, δ-proteobacteria in our study were prevalent throughout the decomposition process, consistent with their suggested importance in the leaching phase of seagrass rhizomes [20] and on surfaces of decomposing eelgrass leaves [29]. Overall, communities were dominated by aerobic chemoorganotrophic taxa typically found on seagrass leaves and/or on macroalgae [84–87]. For instance, the genus *Blastopirellula* (family Pirellulaceae) is a common associate of seagrass and macroalgae and has previously been found in the Baltic Sea [88]. The community composition on decomposing seagrass overlapped with the composition on living seagrass leaves at day 0 here and in previous studies, and includes common surface-attached chemoorganotrophic such as *Granulosicoccus*, *Lewinella* and Rhodobacteraceae, as well as *Methylotenera* that are often in the core microbiome of eelgrass leaves [89]. On day 52, the taxa that took over, such as the genera *Marinomonas* and *Reichenbachiella*, are known for breaking down lignocellulosic material in seagrasses [84] and complex polysaccharides [90], respectively.

We observed a significant shift in the nitrogen-fixing community during the degradation process, from early cyanobacterial dominance to a later dominance by heterotrophic bacteria. For instance, the heterocystous cyanobacterium *Sphaerospermopsis*, which thrives almost exclusively in freshwater habitats [91], dominated the nitrogen-fixing communities during the first 10 days while anaerobic sulphate-reducing bacteria (particularly members of *Desulfopila)*, aerobic (*Insolitispirillum*) and facultative anaerobic (*Shewanella*) found in diverse marine habitats, e.g. tidal-flat sediments [92], dominated later. We speculate that *Sphaerospermopsis* was part of the existing cyanobacterial epiphytic biofilm on live eelgrass leaves, while heterotrophic bacteria, including sulphate reducing taxa, with specific decomposing metabolic capacities proliferated at later stages of the degradation process. A similar succession has been observed in macroalgal detrital systems with sulphate-reducing bacteria fixing nitrogen particularly in dark incubations [26]. Interestingly, despite that the diazotrophs associated with decomposing seagrass appeared phylogenetically distinct, we also observed anaerobic diazotrophic taxa, such as *Desulfopila* and *Malonomonas*. These have previously been found in sediments [93] and were also detected in our sediment samples. Specifically, the sulphate-reducers within the genus *Desulfopila* have been observed in sediments of seagrass ecosystems, contributing to sulphate reduction [75]. The co-occurrence of such taxa on decomposing seagrass and in sediments may be attributed to either sediment input through resuspension or the active participation of certain taxa typically found in anoxic sediments in the degradation of seagrass.

Based on the analysis of *nifH* transcripts, nitrogen fixation measured under light conditions was primarily driven by heterocystous cyanobacteria, particularly *Sphaerospermopsis* and an unknown taxon within the Nostocales order. Although detailed information about the most prevalent Nostoc diazotroph in our *nifH* transcripts was unavailable, it is unsurprising to find a heterocystous nitrogen-fixing cyanobacterium active in marine coastal environments, particularly in association with seagrass, given their common occurrence in symbiotic associations [94]. *Sphaerospermopsis* is known from freshwaters [95] and has to our knowledge not previously been observed associated with seagrass. It is, however, considered an invasive species, potentially driven by phosphorus availability and eutrophication [91]. Hence, the finding of prevalent *Sphaerospermopsis* in seagrass DNA and RNA *nifH* gene transcripts is likely related to the local low saline (salinity < 20) and relatively rich nutrient conditions. The epilithic *Nunduva* genus, a filamentous heterocystous cyanobacterium from the Rivulariaceae family, also contributed substantially to the *nifH* transcripts on seagrass leaf detritus. *Nunduva* sp. forms mats on rocks in intertidal and supratidal marine coastal waters [58, 96]. Interestingly, UCYN-A2 accounted for some of the *nifH* gene expression associated with the decomposing seagrass, and identical sequences were found in the seagrass-associated DNA, as well as in DNA from the surrounding seawater. This cyanobacterial/haptophyte symbiosis has been recovered from coastal areas worldwide [53, 97], including local Danish/Baltic waters [54, 98–100]. This is to our knowledge the first observation of UCYN-A2 being associated with eelgrass, but it has locally been detected associated with copepods [100]. While cyanobacteria dominated the *nifH* gene transcripts, a few putative heterotrophic taxa were also found (Fig. 6).

## Concluding Remarks

In this study, we have shown that distinct microbial taxa thrive on decomposing seagrass leaves and that they undergo a pronounced community succession over time as conditions change during the course of decay. Similarly, a succession in diazotrophs was observed, both in composition and in nitrogen fixation activity. Importantly, our work identifies decomposing seagrass as loci for nitrogen fixation in temperate coastal waters, representing a previously overlooked nitrogen source. Seagrass decay and microbial breakdown processes have been extensively studied in relation to C sequestration in marine environments. However, while nitrogen fixation has been documented in live seagrass leaves in diverse marine environments, including the Mediterranean and tropical waters, our study suggests the need for future research to investigate whether eelgrass debris similarly contributes to nitrogen cycling in these environments, as observed in Danish waters.

## Supplementary Information

Below is the link to the electronic supplementary material.

Supplementary file1 (PDF 957 KB)

## Data Availability

The datasets generated during and/or analysed during the current study are available from the corresponding author on reasonable request.
