# Supplementary material for: Nitrogen Fixation and Microbial Communities Associated with Decomposing Seagrass Leaves in Temperate Coastal Waters
Source: Microb Ecol. 2024 Aug 14;87:106. doi: 10.1007/s00248-024-02424-w (PMC11324715; doi:10.1007/s00248-024-02424-w)
Supplement: Supplementary file 1 [file 248_2024_2424_MOESM1_ESM.pdf]

Supplementary Information (SI)

Nitrogen fixation and microbial communities associated with decomposing seagrass leaves in temperate coastal waters

Vasiliki Papazachariou[1,2], Victor Fernández-Juárez[1], Laura Wegener Parfrey[3], Lasse Riemann[1,2*]

[1]Marine Biological Section, Department of Biology, University of Copenhagen, Helsingør, Denmark
[2]Center for Volatile Interactions, Department of Biology, University of Copenhagen, Copenhagen, Denmark
[3]Biodiversity Research Center, Department of Botany, University of British Columbia, Vancouver, Canada

[*]Corresponding author: Lasse Riemann (email: lriemann@bio.ku.dk)

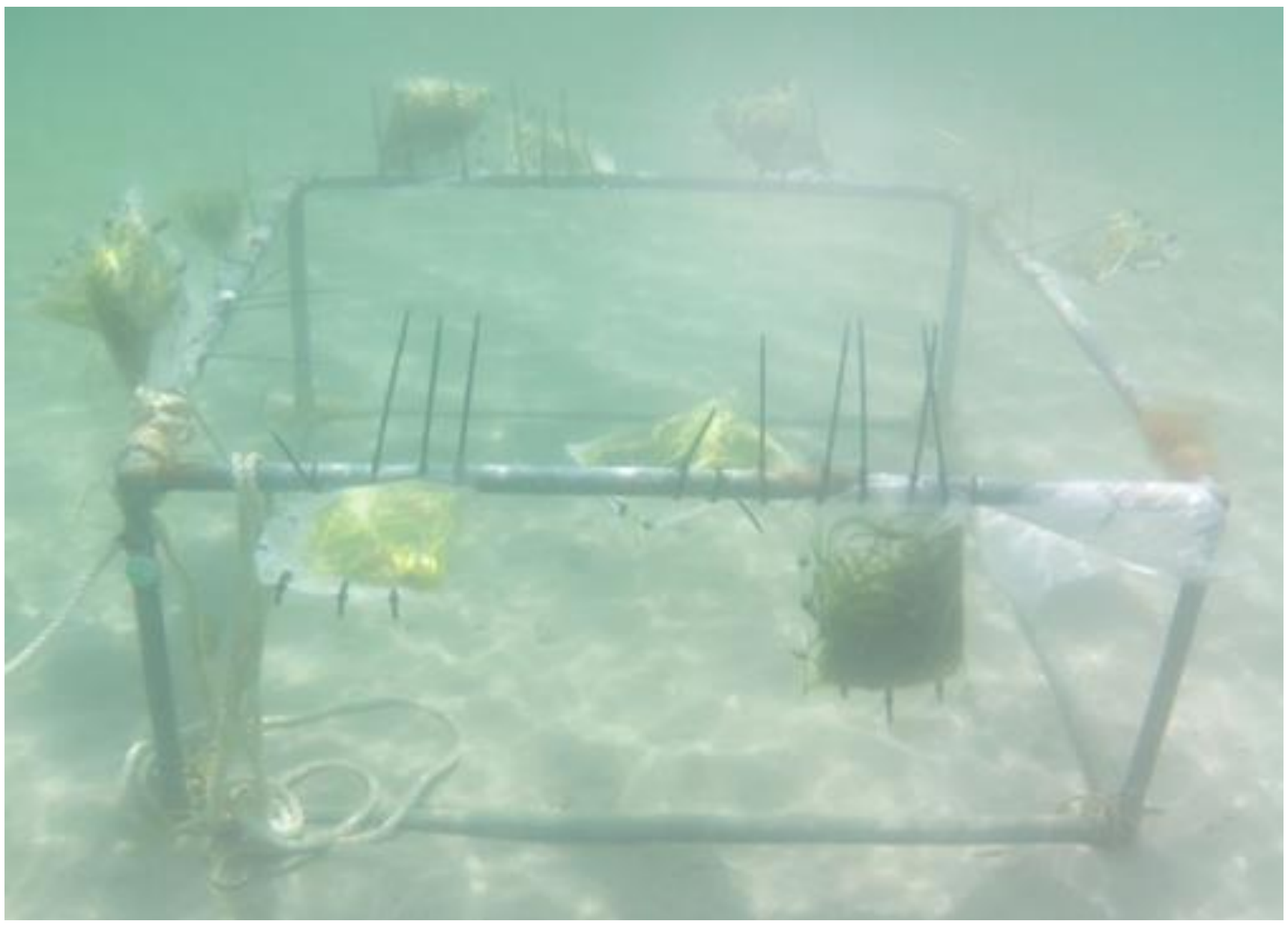

Fig. S1 Metal frame with 1.0 mm polyester mesh bags containing leaves of *Zostera marina*. Depth is ~2 m

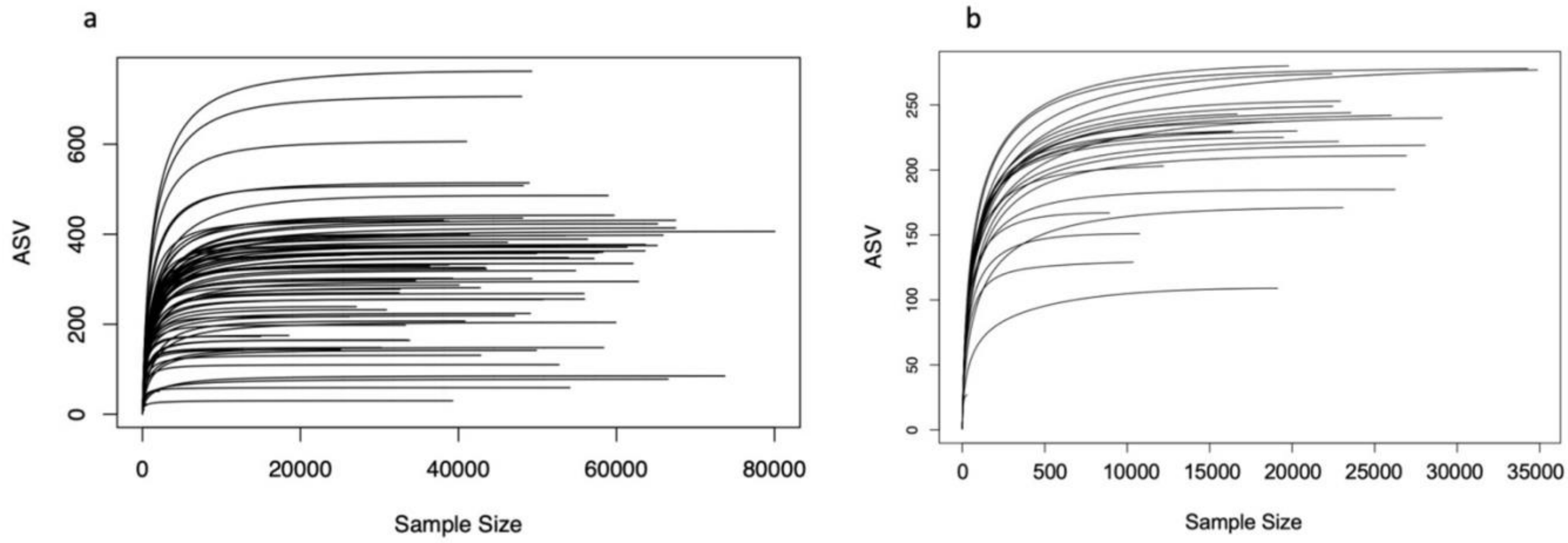

Fig. S2 Rarefaction curves for amplicon sequences (reads) for (a) *nifH* and (b) 16S rRNA genes. ASV: amplicon sequence variants

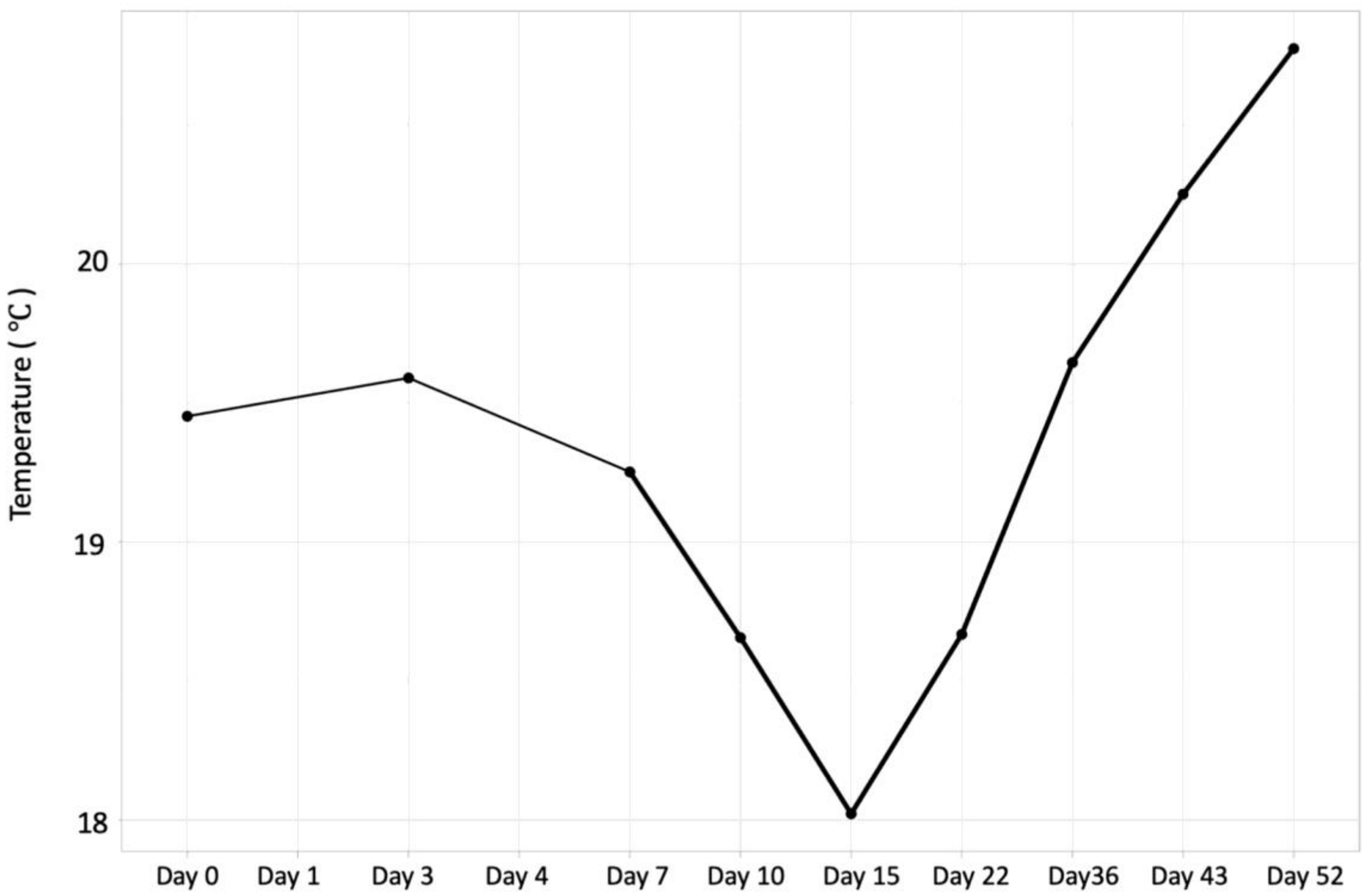

Fig. S3 Seawater temperature during the decomposition experiment

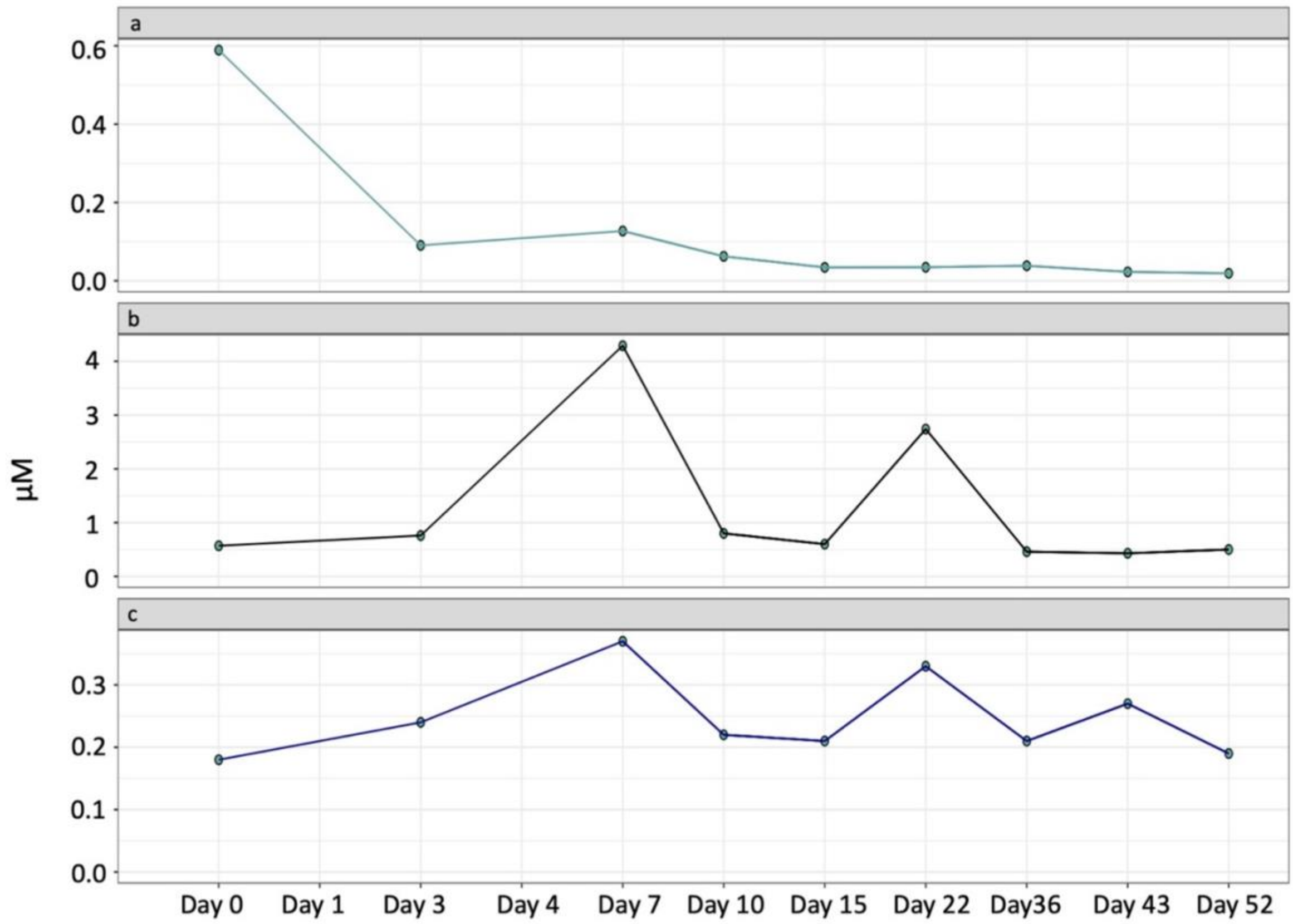

Fig. S4 Inorganic nutrients in the surrounding seawater along the course of decomposition; (a) ammonia (NH_4^+), (b) nitrate (NO_3^{2-}), and (c) phosphate (PO_4^{3-}). Each point represents the mean of duplicate measurements

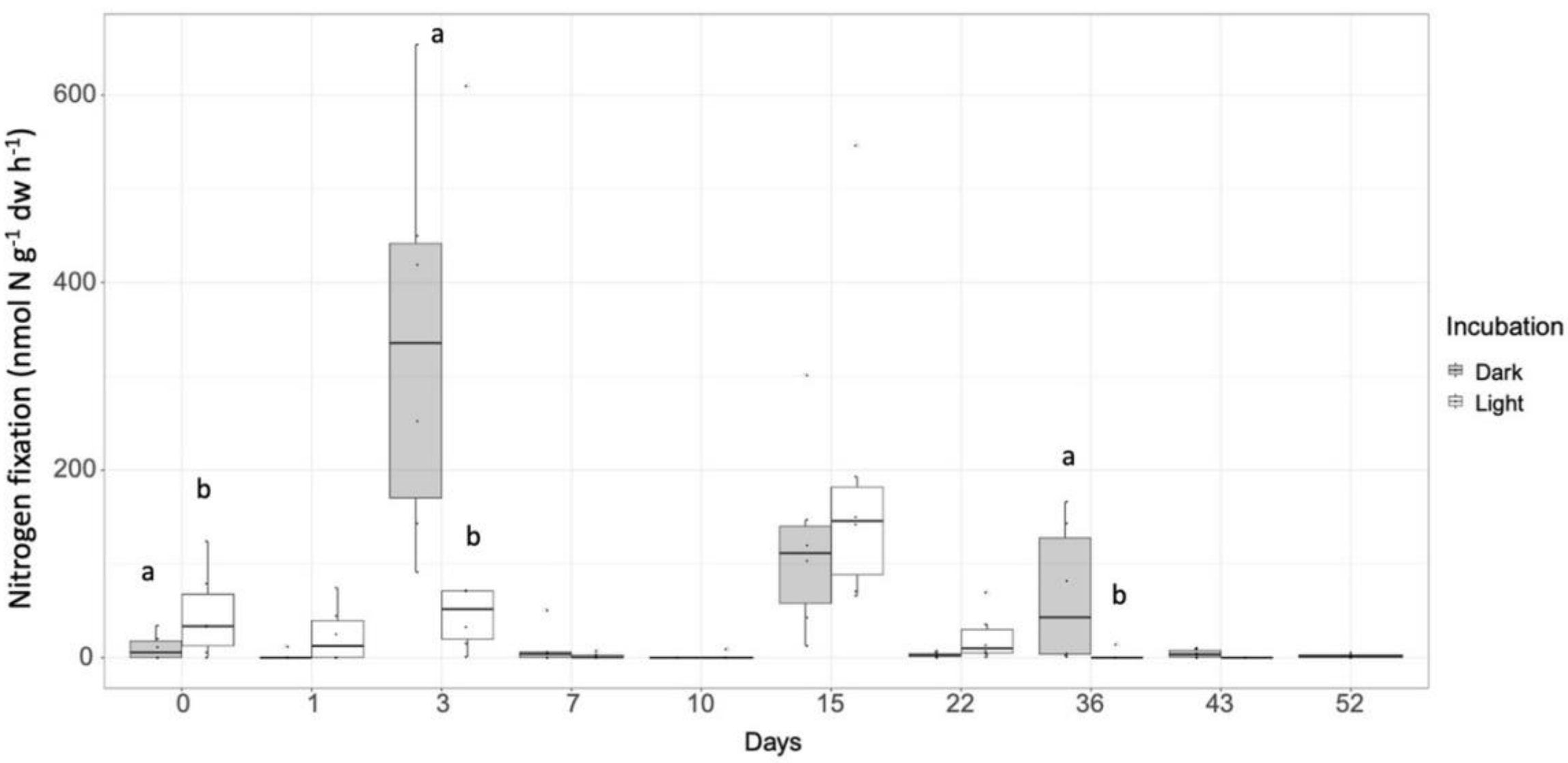

Fig. S5 Nitrogen fixation rates associated with decomposing Z. marina leaves over time. Leaves (n=6) were incubated under dark (dark grey) and light (white) conditions. The lines in the boxes represent the median. Letters above incubations on days 0, 3 and 36 represent statistical difference (P_{adj} <0.05) between means of light and dark incubation per day tested with Kruskal-Wallis followed by a posthoc Dunn's using Bonferroni correction. Only days with significant differences light-dark incubations are shown

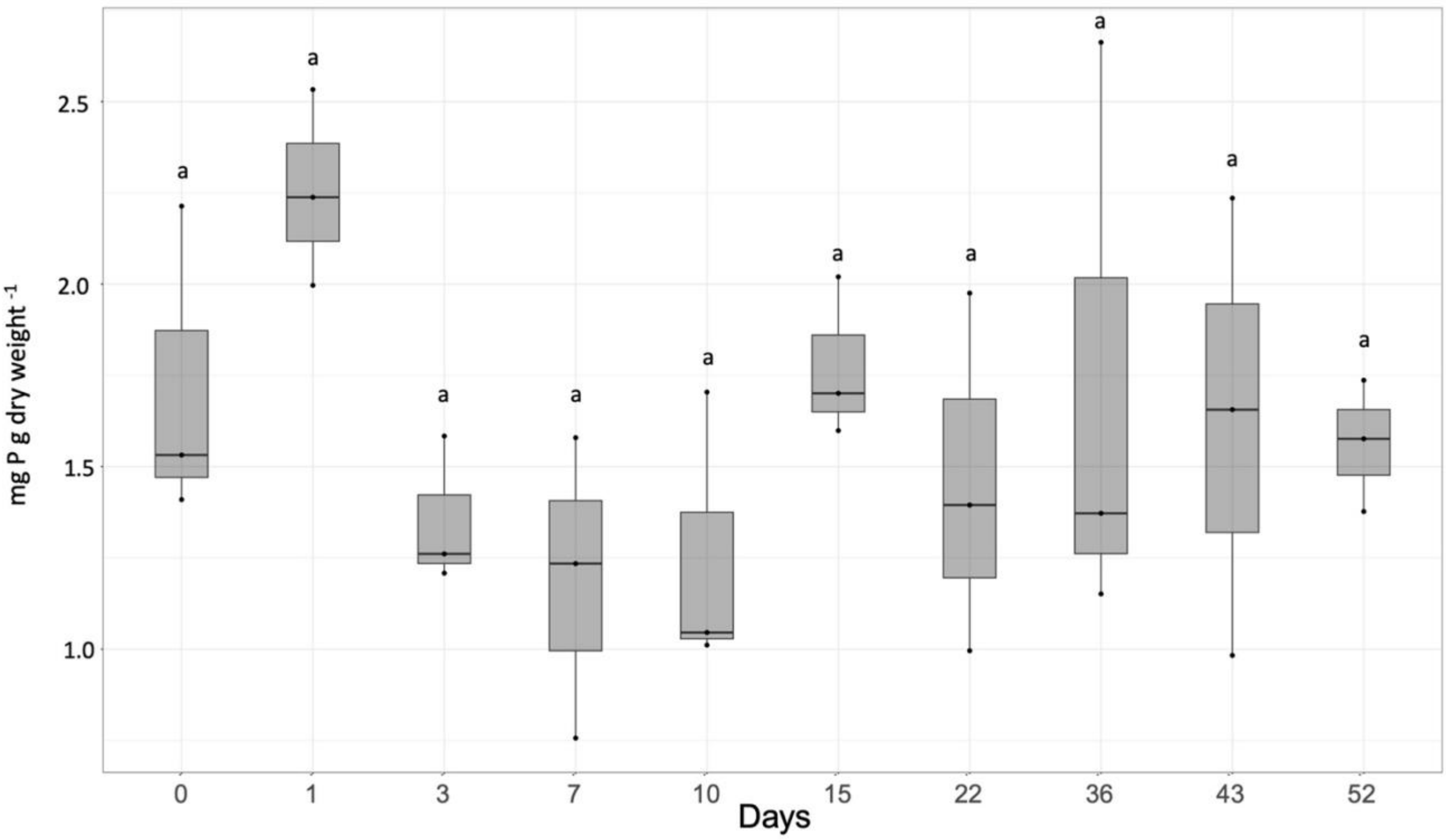

Fig. S6 Elemental phosphorus content in seagrass decomposing leaves. Letters (a, b, and c) represent statistical significance calculated with ANOVA followed by Tukey post-hoc (TukeyHSD). N = 3 per sample

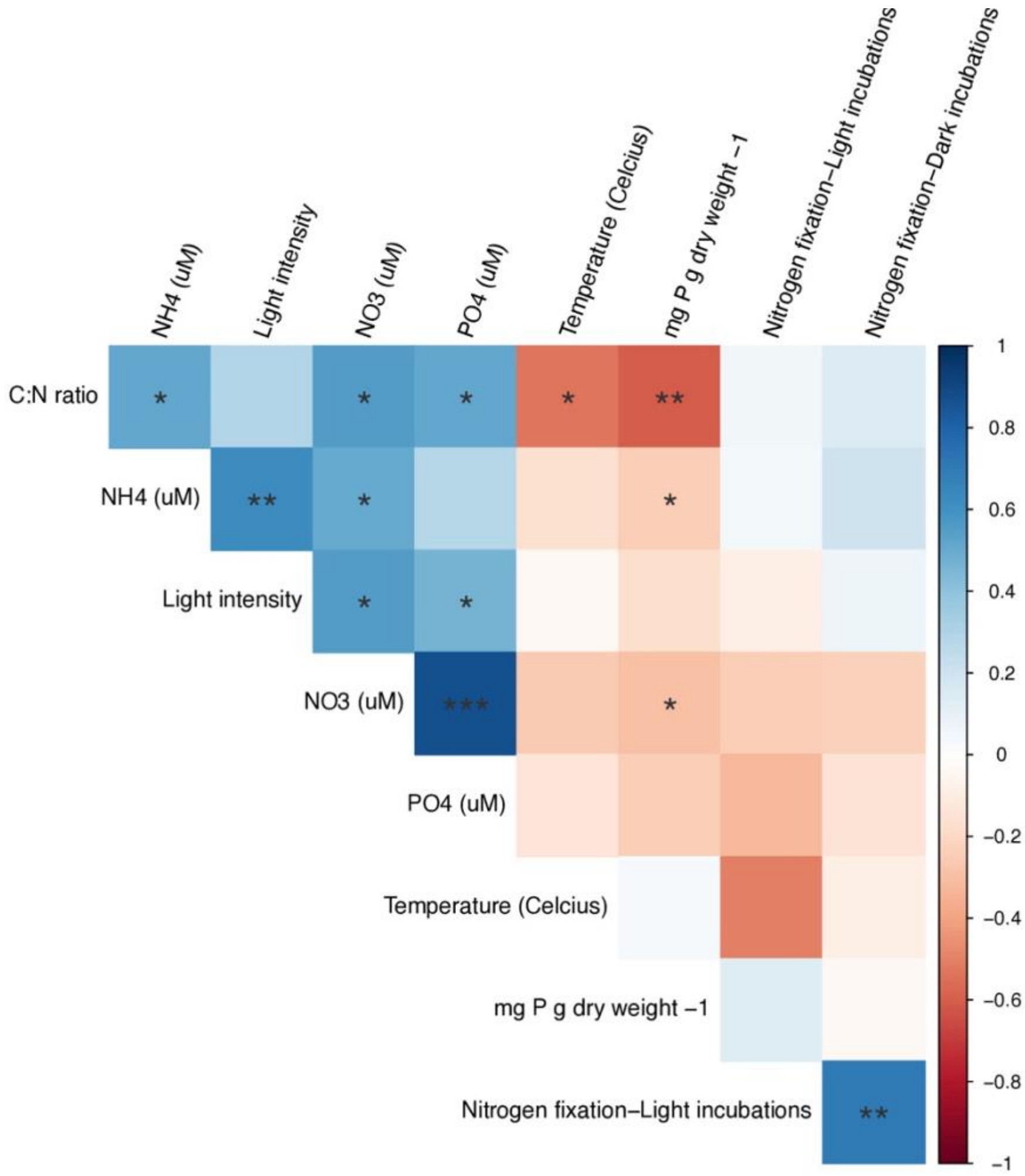

Fig. S7 Pearson correlation coefficients among environmental variables including seawater nutrients (C:N:P leaf elemental content, NH_4^+, NO_3^{2-}, PO_4^{3-}), seawater temperature, light intensity throughout the experiment, nitrogen fixation under light and dark conditions, C:N ratio and P elemental leaf content calculated using the corrplot package in R [1, 2]. Numbers in the boxes represent p-values

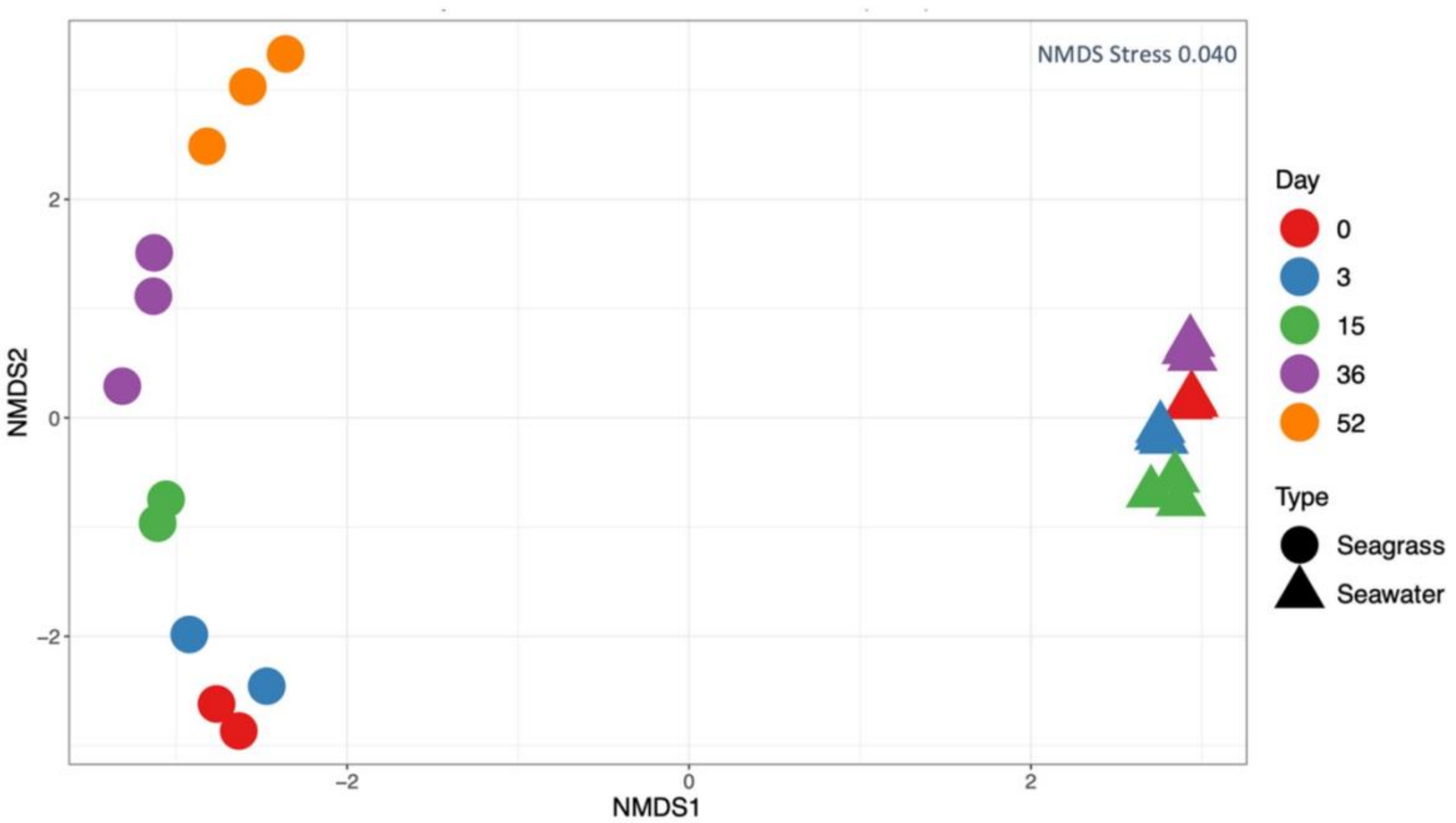

Fig. S8 Non-metric dimensional scaling (NMDS) plot of microbial community composition based on 16S rRNA gene sequences across seagrass and seawater samples over the 52-days decomposition period based on Bray Curtis dissimilarity. Variation between days and between different substrates was tested using permutational multivariate analysis of variance (PERMANOVA) performed by adonis2. This test explained via the percentage (R^2) the variation between substrates (R^2 = 0.45, P =0.001***) and between days (R^2 = 0.31, P =0.01*)

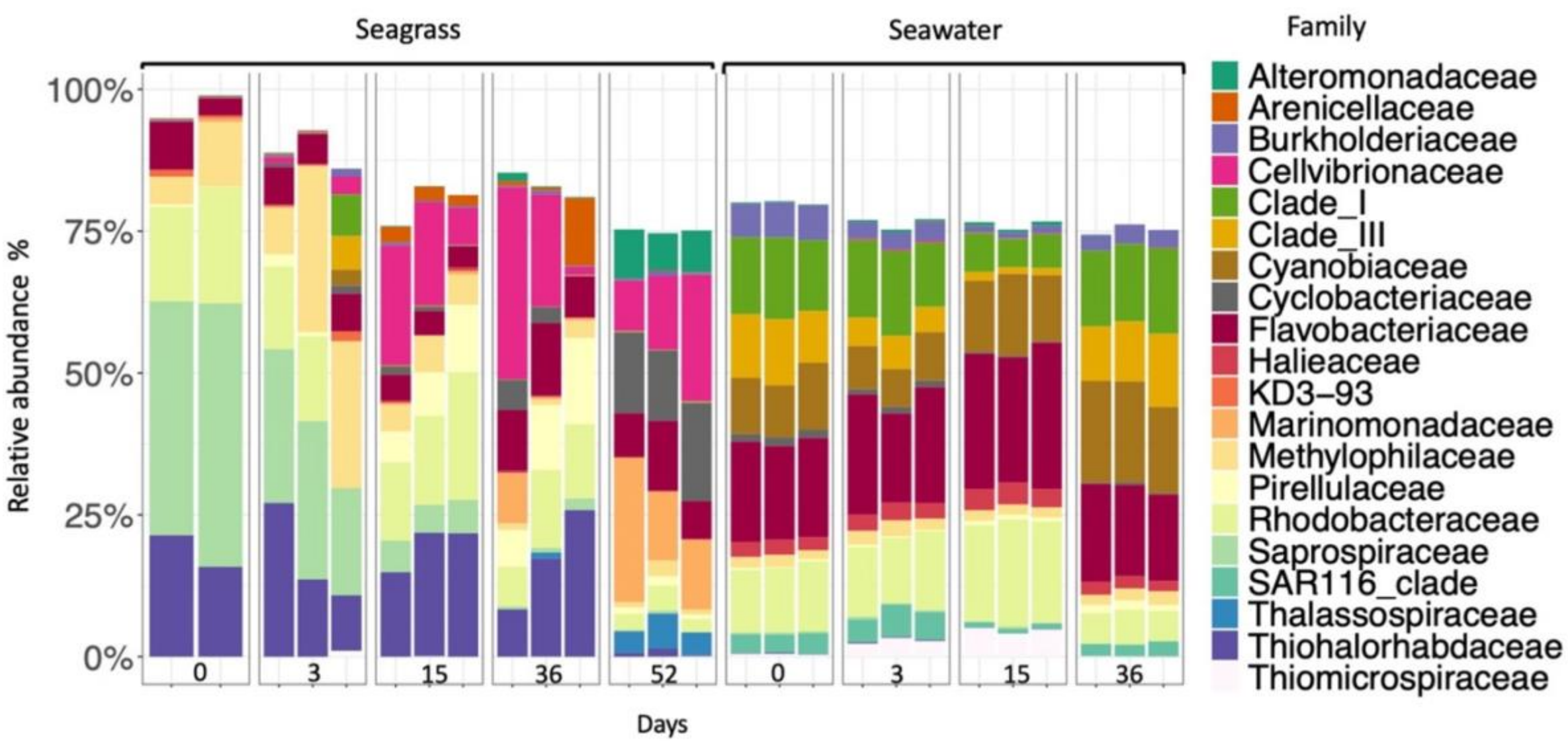

Fig. S9 Microbial community composition on seagrass and in the surrounding seawater at the family level based on 16S rRNA gene sequencing. The plot includes the 20 most abundant families

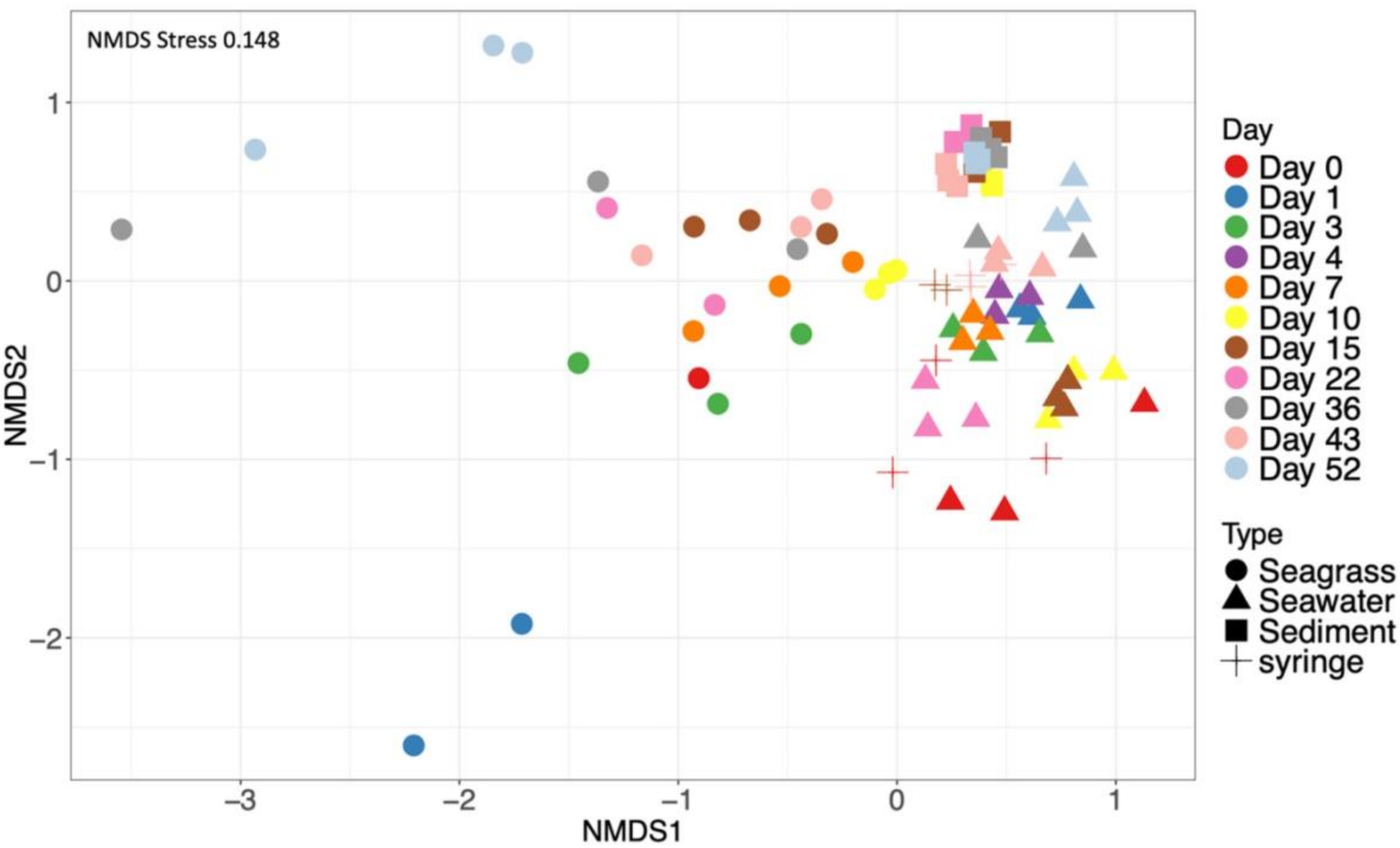

Fig. S10 Non-metric multidimensional scaling (NMDS) plot based on the composition of *nifH* genes amplified from DNA samples along the decomposition based on Bray-Curtis dissimilarity. Days are indicated by colour and substrate by shape

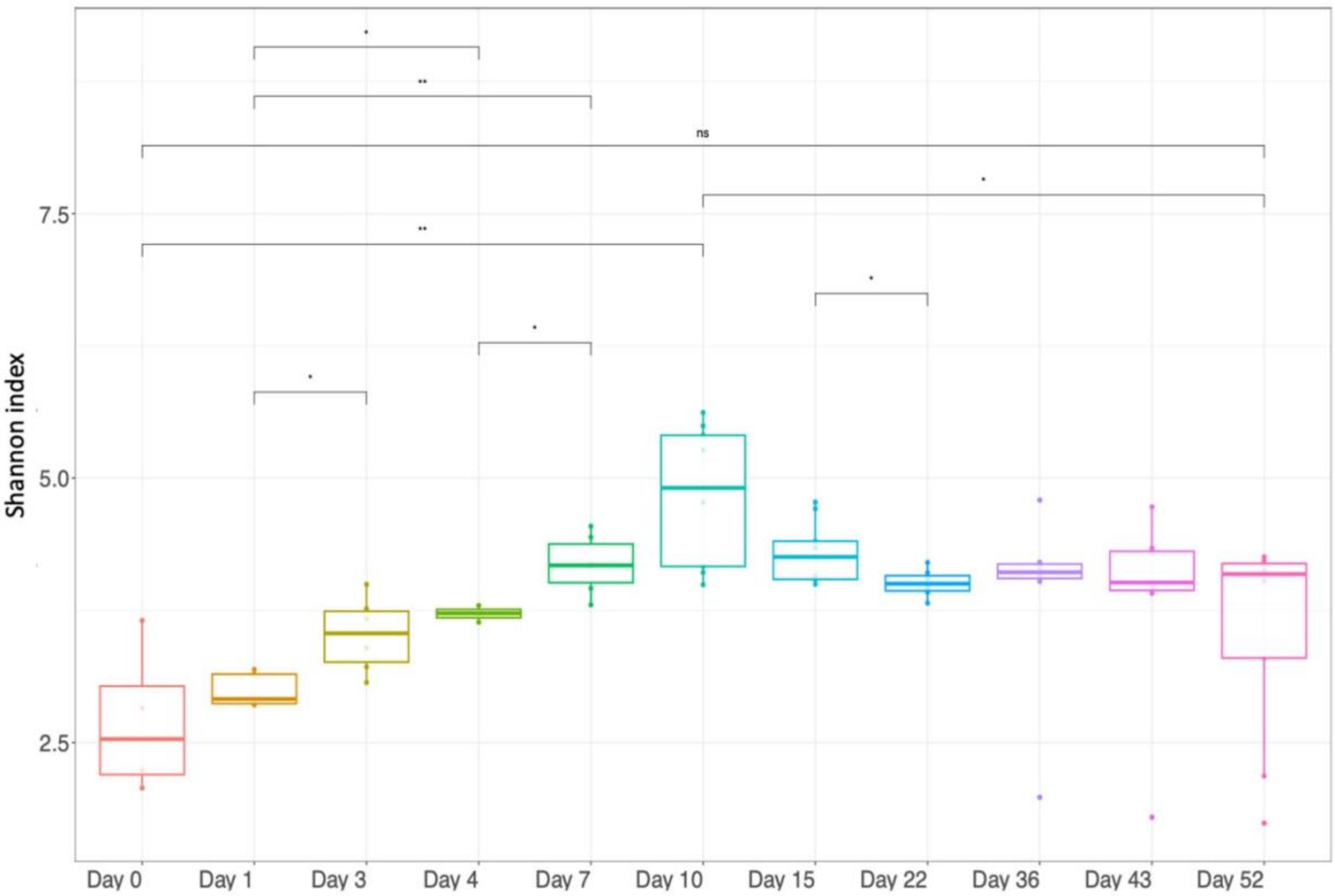

Fig. S11 Alpha diversity of *nifH* genes amplified from DNA over the course of the seagrass decomposition indicated by the Shannon index. Data have been rarefied to even depth (20,000 reads per sample). Asterisks show statistical significance according to a non-parametric Wilcoxon test between days of decomposition (** P =0.001, * P =0.05, ns =not significant)

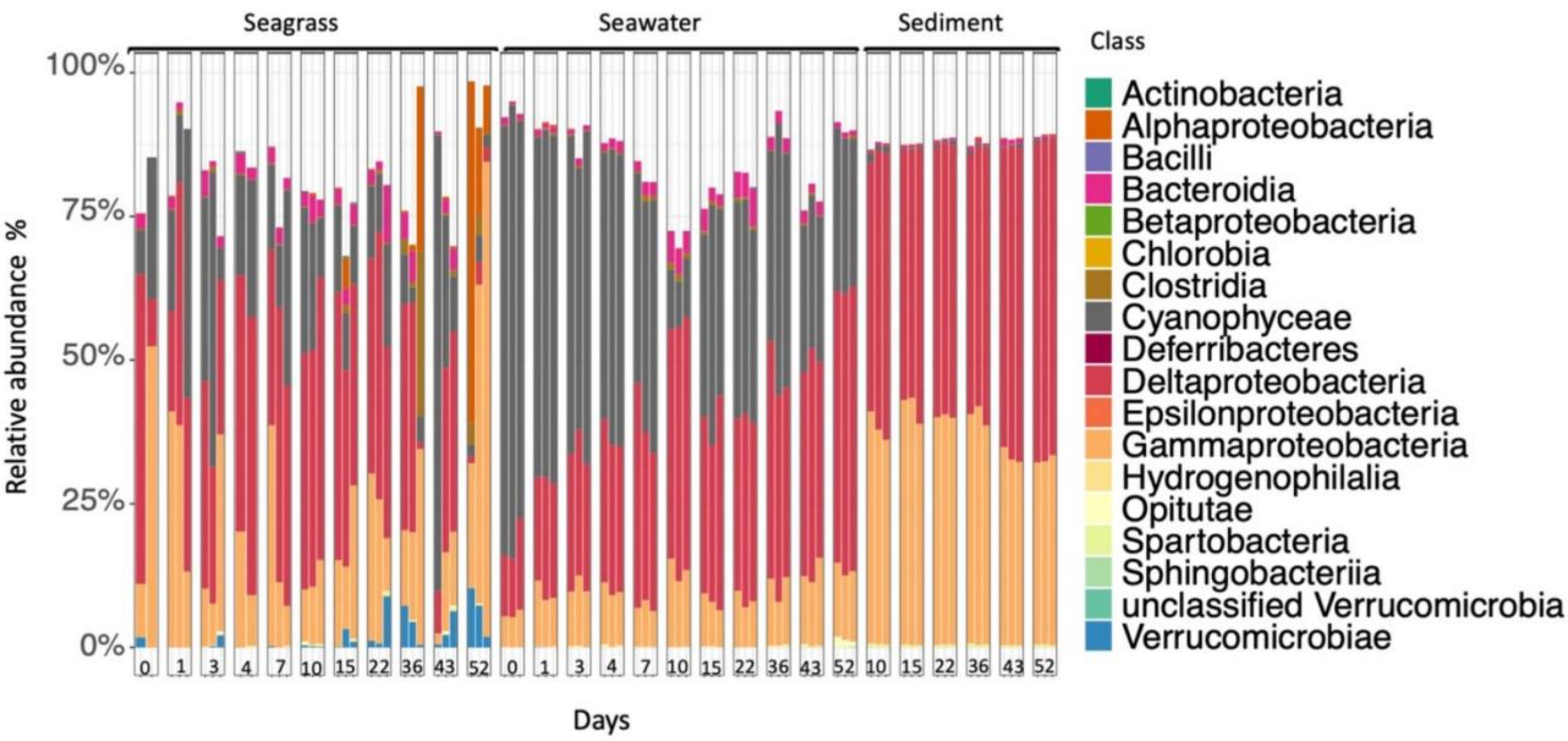

Fig. S12 Relative abundance of diazotrophs over time on decaying seagrass leaves, in the surrounding seawater and in sediments nearby. Based on *nifH* amplicon sequencing of DNA. Each bar represents one replicate. The 20 most relatively abundant taxa are shown. Sediment sampling started on day 10. One seagrass replicate from each of days 0 and 4 was excluded from the analysis due to low read numbers

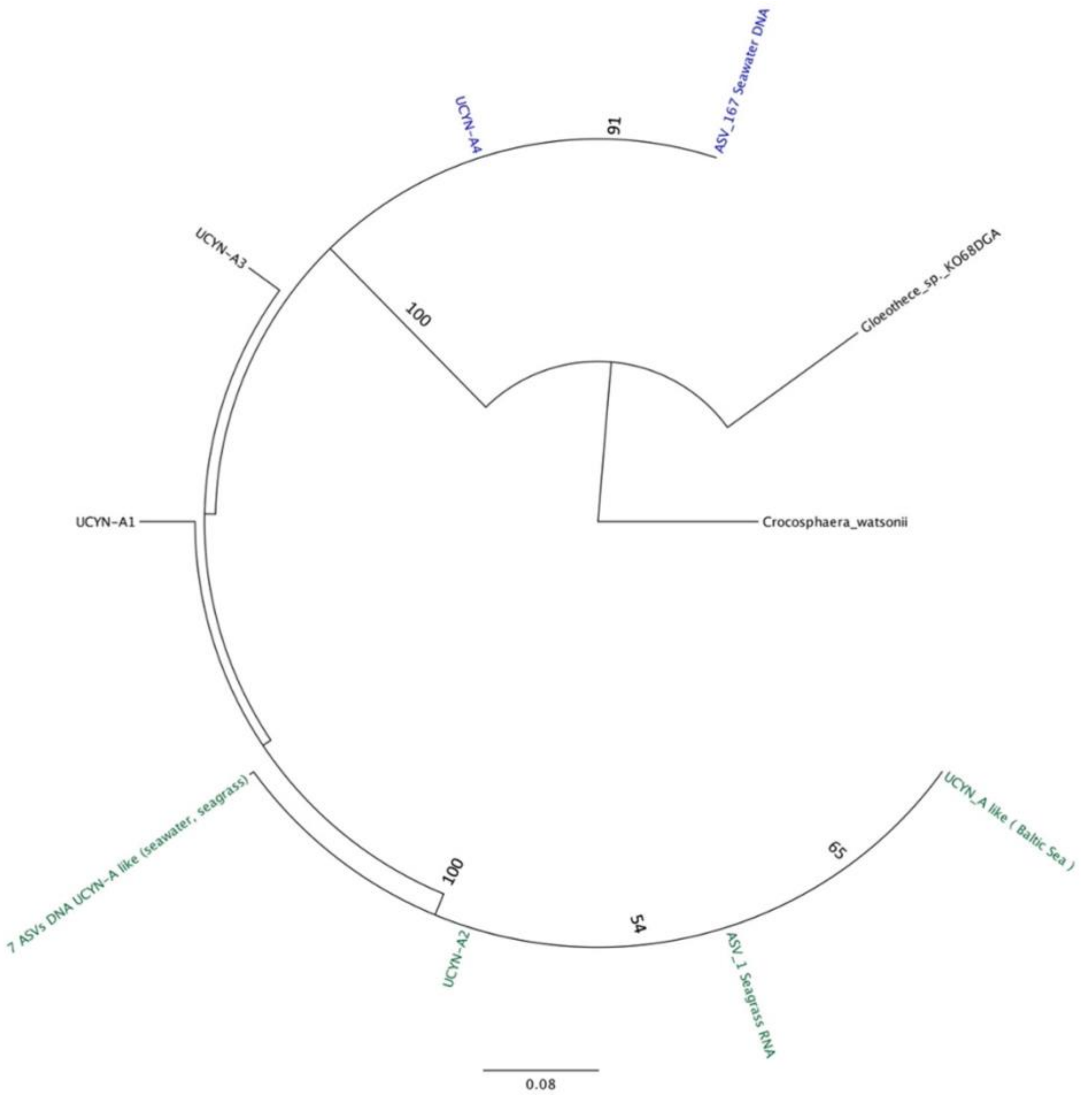

Fig. S13 Maximum-likelihood tree with 1000 bootstraps depicting ASVs related to *Candidatus atelocyanobacterium thalassa* (UCYN) obtained from *nifH* DNA and RNA from seagrass and *nifH* DNA from seawater. Seven ASVs from different samples formed a tight cluster and is for clarity labelled as '7 ASVs DNA UCYN-A like (seawater, seagrass)'. UCYN-A nucleotide reference sequences are from [3, 4]. UCYN-A like (Baltic Sea) nucleotide reference sequence is from [5].

References

1. Friendly M (2002) Corrgrams: Exploratory Displays for Correlation Matrices. Am Stat 56:316–324. https://doi.org/10.1198/000313002533

2. Murdoch DJ, Chow ED (1996) A Graphical Display of Large Correlation Matrices. Am Stat 50:178–180. https://doi.org/10.2307/2684435

3. Farnelid H, Turk-Kubo K, Muñoz-Marín MC, Zehr JP (2016) New insights into the ecology of the globally significant uncultured nitrogen-fixing symbiont UCYN-A. Aquat Microb Ecol 77:125–138. https://doi.org/10.3354/ame01794

4. Turk-Kubo KA, Farnelid HM, Shilova IN, et al (2017) Distinct ecological niches of marine symbiotic N2 - fixing cyanobacterium Candidatus Atelocyanobacterium thalassa sublineages. J Phycol 53:451–461. https://doi.org/10.1111/jpy.12505

5. Salamon Slater ER, Turk-Kubo KA, Hallstrøm S, et al (2023) Composition and distribution of diazotrophs in the Baltic Sea. Estuar Coast Shelf Sci 294:108527. https://doi.org/10.1016/j.ecss.2023.108527
